# Intraspecific Variability of Floral Nectar Volume and Composition in Rapeseed (*Brassica napus* L. var. *oleifera*)

**DOI:** 10.3389/fpls.2016.00288

**Published:** 2016-03-16

**Authors:** Michele Bertazzini, Giuseppe Forlani

**Affiliations:** Department of Life Science and Biotechnology, University of FerraraFerrara, Italy

**Keywords:** nectar production, phloem sap, amino acid and sugar content, nectary metabolism, honeybee preference

## Abstract

Numerous angiosperms rely on pollinators to ensure efficient flower fertilization, offering a reward consisting of nourishing nectars produced by specialized floral cells, known as nectaries. Nectar components are believed to derive from phloem sap that is enzymatically processed and transformed within nectaries. An increasing body of evidence suggests that nectar composition, mainly amino acids, may influence pollinator attraction and fidelity. This notwithstanding, little is known about the range of natural variability in nectar content for crop species. Sugar and amino acid composition of nectar harvested from field-grown plants at the 63–65 phenological stage was determined for a set of 44 winter genotypes of rapeseed, a bee-pollinated crop. Significant differences were found for solute concentrations, and an even higher variability was evident for nectar volumes, resulting in striking differences when results were expressed on a single flower basis. The comparison of nectar and phloem sap from a subset of eight varieties pointed out qualitative and quantitative diversities with respect to both sugars and amino acids. Notably, amino acid concentration in phloem sap was up to 100 times higher than in nectar. Phloem sap showed a much more uniform composition, suggesting that nectar variability depends mainly on nectary metabolism. A better understanding of the basis of nectar production would allow an improvement of seed set efficiency, as well as hive management and honey production.

## Introduction

Many plants require pollinator visitation to obtain efficient seed set. Dicotyledonous species often attract pollinators by offering them a reward of floral nectars. The nectar is a nutrient-rich aqueous solution of sugars, amino acids, organic acids, proteins, fats, vitamins, minerals, and other minor components, such as proteins with high antimicrobial activity (Nicolson and Thornburg, 2007). It is derived from the phloem sap and is produced by a group of specialized cells, the nectaries, usually present in the flower at the base of the petals (De La Barrera and Nobel, 2004). Besides, nectar may also contain secondary metabolites such as terpenes, alkaloids, flavonoids, vitamins, and oils (Truchado et al., 2008). Nectar has a significant metabolic cost for the plant, so as to often be resorbed once fertilization has occurred (Nepi and Stpiczyńska, 2008). Its composition can vary greatly depending on plant species and environmental conditions (Herrera et al., 2006), as well as on floral sexual phases (Antoñ and Denisow, 2014), and flower position within inflorescences (Lu et al., 2015). Total sugar content ranges from a minimum of 5% to a maximum of 80%. In most cases, sucrose is the only or main component, but in some cases sucrose, glucose and fructose are present in similar amounts, conditioning pollinator choice (Lotz and Schondube, 2006). Only rarely other monosaccharides such as raffinose, galactose, and sorbitol are found. Since the phloem sap contains mostly sucrose, chemical reactions must occur to produce glucose and fructose in the nectar. Unequivocal data have been reported showing that these reactions are catalyzed by transglucosidases and transfructosidases localized in the nectaries (Heil, 2011).

Amino acids are also found in nectar but at much lower quantities (typically 0.02–4.8% organic matter), and the biological significance of their presence is still being debated. Some authors have shown that plants pollinated by butterflies contain a higher concentration of amino acids in their nectar than those pollinated by birds (Baker and Baker, 1973). It was believed that amino acid composition was constant in plants of the same species, even if grown in different environments (Baker and Baker, 1977). However, this concept has been superseded by experimental evidence showing a high variability both inter- and intra-populations, strongly influenced by nitrogen availability (Gardener and Gillman, 2001b). The quantity and quality of amino acids in the nectar may enhance insect longevity and fecundity. Radiotracer studies showed that amino acids ingested by adults are incorporated into eggs. Female butterflies prefer nectars spiced with amino acids to nectars lacking them, whereas males show no preference (Mevi-Schutz and Erhardt, 2005).

Among amino acids found in nectars, proline has a unique feature: it is capable of stimulating the labellar salt receptor cells of some insect species, which therefore seem able to recognize the taste (Hansen et al., 1998; Wacht et al., 2000). Nectar foraging insects preferentially utilize proline during the initial phases of flight (Micheu et al., 2000). The availability of an energetic substrate ready to be used and suitable for intense flight phases can represent an advantage for bees during long distance foraging. Proline represents the most abundant amino acid in the haemolymph of many insects, including honeybees (Crailsheim and Leonhard, 1997), and high amounts of proline are found in many types of nectar. In tobacco plants it can accumulate to levels of 45–60% of total amino acids (Carter et al., 2006). In addition to proline, aromatic amino acids (tyrosine, phenylalanine), serine, and amides (glutamine, asparagine) may also be present at high concentrations (Gardener and Gillman, 2001a). Increasing evidence supports the preference of bees and butterflies for sugar solutions enriched with proline. The concentration range preferred by honeybees (from 2 to 6 mM; Carter et al., 2006) is close to that found in several natural nectars (Gardener and Gillman, 2001a). Such a preference does not seem to exist in bird pollinators (Leseigneur et al., 2007). This suggests a co-evolutionary strategy for increasing pollination of plants that produce proline-rich nectar by foraging insects that perceive its presence (Biancucci et al., 2015). Our earlier studies found a strong preference of proline-rich nectar and aversion to nectar containing serine by forager honeybees (Bertazzini et al., 2010), and these preferences and aversions may influence the frequency of flower visitation by insects.

Similarly, the quality and the ratios between various types of sugars and their absolute amounts *per* flower may greatly alter the attractiveness to pollinators. In the case of honeybees, it is well-known that the profitability of a flower, defined as the ratio between the caloric cost to fly to and visit a single flower and the mean caloric gain that can be obtained while foraging, is one of the main determinants of flower choice and of dance communication of scout honeybees to hivemates (Waddington, 1982). Amino acid and sugar content may thus contribute in providing the basis for flower constancy, the phenomenon by which an individual forager actually bypasses rewarding flowers to restrict visits to a single plant species (Grüter and Ratnieks, 2011). A better knowledge of these aspects may open new perspectives in both hive management and optimization of crop yield. The occurrence of a natural variability in nectar composition among cultivars of a bee-pollinated crop could cause a different frequency of visit by pollinators, resulting in different seed set efficiency, and significantly influencing final grain harvest. Moreover, positioning hives near a field where a preferred nectar-producing crop variety is cultivated could *persuade* the bees to visit this source of nectar. Feeding on a single plant species, bees would produce a more valued (*unifloral*) honey, with a distinctive aroma and flavor.

Many crops are dependent to different degrees on honeybees for pollination. These include apples, avocados, cherries, cranberries, sunflowers, alfalfa, cucumbers, kiwi fruit, and melons (Ball, 2007). Oilseed rape (*Brassica napus* L. var. *oleifera*), despite being considered a predominantly wind-pollinated and self-compatible plant, in a number of studies showed significantly increased grain yields when bee-pollinated (Chambó et al., 2014, and the references therein). Winter rapeseed cultivation has dramatically increased in Europe following the 2003/30 Directive of the European Parliament promoting the use of biofuels to replace diesel or gasoline for transport. Almost one-third of the total cropped area has been reported to be entomophilous, and pollinators not only double the final yield, but also contribute to achieve uniform and early pod setting (Abrol, 2007). Several studies investigated sugar composition of rapeseed nectar (reviewed in Westcott and Nelson, 2001). In most cases sucrose was present at very low levels, together with high fructose concentrations (e.g., Kevan et al., 1991). The presence of a low fructose-to-glucose ratio in unifloral rapeseed honey is the cause of its high tendency to granulate, a property that forces beekeepers to harvest it as soon as it is capped. After collecting, it has to be extracted within 24 h and marketed within a few weeks. Despite these unfavorable features, rapeseed shows one of the highest melliferous potentials and represents a main forage crop for bees. Many palynologic analyses showed the presence of notable percentages of rapeseed pollen in multifloral honeys (e.g., Sabo et al., 2011). Yet, a recent calculation of the theoretical maximal honey yield revealed that this bee pasture may be considerably underutilized (Nedić et al., 2013).

The occurrence of a significant natural variability in sugar and amino acid content in nectar would allow breeding programs to increase both the attractiveness to honeybees (influencing in turn crop yield) and honey quality. With the aim to confirm previous data on sugar content and obtain new information on amino acid composition, we harvested and analyzed nectars from a large group of rapeseed varieties. To investigate the relative role of phloematic sap and nectary metabolism in establishing the nature and quantity of sugars and amino acids in floral nectars, the phloem sap of a subset of genotypes was also analyzed.

## Materials and methods

### Plant growth

Seeds of 44 commercial winter cultivars of *B. napus* L. var. *oleifera* Metzger (including 16 inbred lines and 28 hybrid varieties, as detailed in Table 1) were sown on October 3^rd^, 2009, in an experimental field located near Jolanda di Savoia (FE), Italy, at 44°54′42.3″ N–11°58′44.4″ E, which during the previous year had not been cultivated. Soil properties were as summarized in Table S1. A completely randomized design with four replicates was adopted (Figure S2). The field was divided into four parts. Each part enclosed 44 plots (2 × 2 m), each one consisting of seven rows, 33 cm apart. A 1.0 m edge was left all around. Seeds were sown with a mechanical planter (1001-B Precision Garden Seeder, Earthway, equipped with a 1002/05 seed plate), obtaining a density of about 50 seedlings m^−1^. Fertilization consisted of 40 kg ha^−1^ N (urea) and 5 kg ha^−1^ P_2_O_5_ (superphosphate) in pre-emergence, and 80+40 kg ha^−1^ N (ammonium nitrate) topdressed at the flower-bud-visibility stage. Irrigation was not used, and no chemical treatment was given in order to limit weed growth. Weeds were removed manually, when required. Immediately before nectar sampling, three plots with plants showing a uniform growth were selected for each rapeseed variety (Figure S2), whereas plants in the fourth plot were not used. Meteorological data (irradiance, temperature, rainfall, relative humidity, and pressure) for the study period are reported in Figure S3.

**Table 1:** **Rapeseed varieties used in this study**.

**Cultivar**	**Source^a^**		**Cultivar**	**Source^a^**		**Cultivar**	**Source^a^**	
Adam	1	Inbred	Gamin	9	Hybrid	Pelican	3	Hybrid
Alpaga	2	Inbred	Henry	10	Inbred	Pr45d01	12	Hybrid
Aragon	3	Inbred	Hercules	4	Hybrid	Pr45d03	12	Hybrid
Avenir	2	Hybrid	Hybristar	8	Hybrid	Pr46w10	12	Hybrid
Bambin	2	Hybrid	Intense	2	Hybrid	Pr46w31	12	Hybrid
Belcanto	2	Hybrid	Katabatic	11	Inbred	Shakira	10	Inbred
Beluga	2	Inbred	Kompass	1	Hybrid	Taurus	3	Hybrid
Dante	4	Inbred	Lorenz	3	Inbred	Toccata	11	Hybrid
Elan	5	Hybrid	Mendel	3	Hybrid	Totem	2	Inbred
Es Artist	6	Hybrid	Milena	5	Inbred	Vectra	4	Hybrid
Exagone	7	Hybrid	Nk Caravel	9	Hybrid	Verona	13	Inbred
Excalibur	7	Hybrid	Nk Formula	9	Hybrid	Viking	3	Inbred
Excel	7	Hybrid	Nk Petrol	9	Hybrid	Zeruca	2	Inbred
Facile	3	Hybrid	Nk Technic	9	Hybrid	Zoom	2	Inbred
Forza	8	Inbred	Palmedor	2	Hybrid			

a *1 Deutsche Saatveredelung Lippstadt, courtesy of Venturoli Sementi*.

*2 Serasem, courtesy of Florisem Italia*.

*3 Norddeutsche Pflanzenzucht Lembke Semences, courtesy of F.lli Moretti Cereali and Florisem*.

*4 RAPS GBR Saatzucht Lundsgaard, courtesy of Carla Sementi*.

*5 KWS SAAT AG, courtesy of Fondazione per l'Agricoltura F.lli Navarra*.

*6 Euralis Semences International, courtesy of Fondazione per l'Agricoltura F.lli Navarra*.

*7 Dekalb—Monsanto Company, courtesy of Monsanto Italia*.

*8 SCA Adrien Momont et Fils, courtesy of Istituto Sementi e Tecnologie Agroalimentari*.

*9 Syngenta Seeds, courtesy of Società Italiana Sementi and NK Sementi Syngenta*.

*10 Intersaatzucht Donau GMBH & Co., courtesy of Padana Sementi Elette*.

*11 Mick Pickford, courtesy of Maisadour Semences Italia*.

*12 Pioneer Hi-Bred, courtesy of Pioneer Hi-Bred Italia*.

*13 Svalof Weibull Ab, courtesy of Padana Sementi Elette*.

*Underlined varieties were also used to harvest phloem sap*.

#### Nectar sampling

Flowers were sampled from plants at the 63 (30% of flowers on main raceme open) to 65 (50% flowers on main raceme open, older petals falling) phenological stage in the BBCH-scale (Lancashire et al., 1991). Nectar was extracted by centrifugation with the method of Bosi (1973). For each sample, 40 freshly-opened flowers were harvested, one flower *per* plant, taking care to discard those already visited by foraging insects. Flowers were transferred into sterile 50-mL centrifugal tubes containing 10 g of acid-washed glass beads (5 mm diameter, Sigma 18406) wrapped in a nylon mesh and covered by a piece of hydrophobic cotton to avoid sample contamination by pollen. Following centrifugation at RT for 3 min at 1000 g, beads and cotton were removed and the nectar was harvested, measured with a micropipette (0.5–10 μL) and transferred into 0.5-mL Eppendorf vials. Samples were immediately frozen and stored at −20°C until the analysis. Nectars were harvested in the afternoon (from 1.30 to 5.30 p.m.), and harvesting was carried out during 4 different days, on April 21^st^, April 25^th^, April 27^th^, and April 29^th^, 2010. Each time, three replications were carried out for a given genotype, one *per* part. Overall, 12 nectar samples were harvested for each rapeseed variety (3 replications [part] × 4 harvest days).

#### Phloem sampling

Phloem sap was harvested with a validated protocol for species belonging to the genus *Brassica* (Giavalisco et al., 2006). Samples were obtained by making small punctures with a sterile hypodermic needle into inflorescence stems of rapeseed plants. The first exuding droplet was discarded, and the subsequent exudate was collected, immediately frozen and stored at −20°C until the analysis. Sampling was carried out in triplication on April 29^th^ for a subset of eight genotypes, which are underlined in Table 1.

### Sugar analysis

Glucose, fructose, and sucrose from nectar were determined enzymatically (Figure S4). To measure glucose concentration in nectars, sample aliquots (0.1 and 0.2 μL) were incubated at 37°C in a final volume of 1 mL with 0.75 mM NAD^+^, 0.5 mM ATP, and 25 mU of both hexokinase and glucose-6-phosphate dehydrogenase (Sigma G3293) in 25 mM Tris-HCl buffer, pH 7.8. The increase in absorbance at 340 nm was followed in a Peltier-equipped spectrophotometer for 15 min, until it stabilized. Fructose content was then quantified by adding 600 mU of phosphoglucose isomerase (Sigma F2668) in a volume of 10 μL. The resulting increase in absorbance was measured for a further 21 min. Sucrose concentration in the same sample was lastly evaluated by adding 30 U of baker yeast invertase (Sigma I4504) in a volume of 10 μL, monitoring the absorbance for a further 24 min. Sugar content was calculated on the basis of calibration curves obtained with suitable dilutions of an artificial nectar composed of 7.5% (w/v, 416 mM) of both glucose and fructose, and 1% (w/v, 29.2 mM) sucrose. In the case of phloem sap, 0.1 and 0.2 μL were analyzed for glucose and fructose, whereas 0.01 and 0.02 μL were used to measure sucrose content, and concentrations were estimated from calibration curves obtained with an artificial phloem sap composed of 0.5% (w/v, 27.8 mM) of both glucose and fructose and 20% (w/v, 584 mM) sucrose.

### Amino acid analysis

Total amino acid content was determined by its reaction with *o*-phthaldialdehyde (*o*PDA) as described previously (Jones et al., 2002), with minor modifications. Sample aliquots (1 and 2 μL) were water-diluted to 50 μL, and the resulting samples were mixed with the same volume of *o*PDA solution (0.5 M in 0.5 M sodium borate buffer, pH 10.0, containing 0.5 M β-mercaptoethanol, and 10% [v/v] methanol). After exactly 60 s, the increase in absorbance was measured at 340 nm using a UV-transparent cuvette with 10 mm optical path (UVette, Eppendorf) and a universal adapter. Amino acid content was extrapolated from a calibration curve obtained with a solution of all the 20 proteinogenic compounds (each at 1 mM but asparagine, glutamic and aspartic acid at 2 mM, and glutamine at 5 mM; Figure S5).

Single amino acids were quantified by RP-HPLC following derivatization with *o*PDA, as described (Forlani et al., 2014). Peaks were integrated by area, with variation coefficients ranging from 0.8 to 3.2%. Since *o*PDA does not react with proline, its concentration was measured either by the acid ninhydrin method (Williams and Frank, 1975), or by RP-HPLC following derivatization with 4-dimethyl-aminoazobenzene-4′-sulfonyl chloride (DABS-Cl). In the latter case, 10-μL aliquots of suitable sample dilutions were mixed with the same volumes of a 0.2 M sodium bicarbonate buffer, pH 10.0, and a 2 mg mL^−1^ solution of DABS-Cl in acetone. After 30 min incubation at 70°C, 20 μL of derivatized samples were injected into a 4.6 × 250 mm Zorbax ODS column (Rockland Technologies, Newport, DE) equilibrated with 65% solvent A (17 mM potassium phosphate buffer, pH 6.5, containing 2% [v/v] dimethylformamide), and 35% solvent B (80% methanol containing 4% [v/v] dimethylformamide). Elution proceeded at a flow rate of 60 mL h^−1^ using a computer-controlled (Data System 450; Kontron, Munchen, Germany) complex gradient from 35 to 95% solvent B, monitoring the eluate at 436 nm. Two technical replications were performed for each sample. For all rapeseed varieties, single amino acid content was determined in a sample obtained by combining the same volume of the 12 existing samples. For a subset of eight genotypes, for which larger nectar volumes were available, four samples were analyzed, each one consisting of a mixture of all three nectar samples harvested in the same day. Phloem saps were analyzed individually.

### Statistical analysis

For ANOVA and Tukey's *post-hoc* HSD analysis, the *Statistica* software package (version 7.1, StatSoft, Tulsa, OK, U.S.A.) was used. Correlation analysis and descriptive statistics were computed with the *Prism 6* software (version 6.03, GraphPad Software, Inc., San Diego, CA, U.S.A.).

## Results

### A great variability is evident among rapeseed genotypes regarding floral nectar volume

Nectar was harvested from plants of 44 rapeseed cultivars, comprising both hybrids, and inbred lines. Preliminary attempts showed that in all cases insect-visited flowers contain negligible residual amounts of nectar (< 1μL in 40 flowers). Therefore, only freshly-opened, unvisited flowers were collected, whose opening diameter was smaller and easily distinguishable from those already visited by honeybees. Moreover, harvesting was carried out in the early to mid-afternoon, when the relative humidity was lower (Figure S3), to exclude contamination by dew and to allow the attainment of steady-state nectar production, which is dependent on the establishment of full photosynthetic rate and phloem loading. The results showed a highly significant (*P* = 0.000000) difference among cultivars with respect to nectar volume, which ranged from 20 to 750 nL flower^−1^ (Table 2).

**Table 2:** **Volume, sugar, and amino acid content of nectars from rapeseed varieties**.

**Cultivar**	Volume ^*^(μL flower^−1^)	**Glucose ^§^ (mM)**	**Fructose ^§^ (mM)**	**Sucrose ^§^ (mM)**	**Amino acids ^#^ (mM)**
Adam	0.51±0.12^i-n^	415±26^b-g^	338±24^b-j^	23±1^a-c^	3.79±0.54^a-e^
Alpaga	0.12±0.02^a-g^	796±56^j^	679±40^l^	29±2^a,g^	4.76±1.83^a-e^
Aragon	0.10±0.02^a-f^	441±44^b-i^	338±31^b-j^	46±3^j-o^	7.02±1.60^a-e^
Avenir	0.14±0.03^a-h^	454±22^c-i^	365±19^d-j^	25±1^a-d^	4.35±1.48^a-e^
Bambin	0.15±0.04^a-h^	409±8^b-g^	345±8^c-j^	25±1^a-d^	7.38±2.01^c-e^
Belcanto	0.16±0.02^a-h^	578±56^i^	428±38^j,k^	42±4^h-n^	3.55±0.85^a-e^
Beluga	0.15±0.02^a-h^	481±12^e-i^	410±8^h-k^	23±1^a-c^	4.62±1.36^a-e^
Dante	0.24±0.06^a-i^	334±10^a-d^	280±11^a-f^	18±1^a^	4.53±1.10^a-e^
Elan	0.12±0.03^a-g^	373±33^b-f^	265±23^a-e^	40±3^g-m^	1.32±0.17^a,b^
Es Artist	0.40±0.08^f-m^	445±35^b-i^	343±25^b-j^	48±3^k-o^	1.96±0.38^a-c^
Exagone	0.39±0.06^d-m^	527±35^g-i^	327±27^b-j^	46±2^j-o^	3.55±0.58^a-e^
Excalibur	0.31±0.06^a-j^	529±8^g-i^	365±10^d-j^	54±4^n,o^	2.84±0.48^a-c^
Excel	0.27±0.04^a-i^	563±17^h,i^	337±13^b-j^	40±1^f-m^	1.34±0.15^a,b^
Facile	0.20±0.04^a-i^	530±9^g-i^	481±5^k^	34±3^c-j^	7.54±1.19^c-e^
Forza	0.68±0.12^m,n^	454±13^c-i^	404±11^h-k^	19±2^a,b^	2.90±0.98^a-d^
Gamin	0.07±0.02^a-e^	401±22^b-g^	266±18^a-e^	43±1^i-n^	7.17±0.66^b-e^
Henry	0.66±0.14^l-n^	412±29^b-g^	334±19^b-j^	22±3^a-c^	3.60±0.84^a-e^
Hercules	0.11±0.03^a-g^	391±13^b-g^	315±11^b-j^	32±2^b-i^	4.58±1.28^a-e^
Hybristar	0.39±0.06^e-m^	481±13^e-i^	409±15^h-k^	25±1^a-d^	0.98±0.18^a^
Intense	0.06±0.02^a-c^	384±14^b-f^	328±12^b-j^	25±1^a-d^	6.08±1.69^a-e^
Katabatic	0.06±0.02^a-c^	374±13^b-f^	319±7^b-j^	26±2^a-e^	2.48±0.78^a-c^
Kompass	0.21±0.03^a-i^	441±10^b-i^	354±9^d-j^	23±2^a-c^	5.34±1.24^a-e^
Lorenz	0.64±0.09^k-n^	309±11^a,b^	231±2^a,b^	38±1^e-l^	2.61±0.60^a-c^
Mendel	0.34±0.05^a-k^	400±10^b-g^	299±7^b-h^	29±2^a-h^	5.46±1.71^a-e^
Milena	0.43±0.08^g-m^	447±54^b-i^	320±36^b-j^	39±3^f-m^	6.17±1.16^a-e^
Nk Caravel	0.35±0.05^b-l^	512±11^f-i^	388±11^f-k^	39±2^f-m^	2.83±0.60^a-c^
Nk Formula	0.09±0.01^a-f^	472±20^d-i^	369±18^e-k^	56±3^o^	8.84±3.00^d-e^
Nk Petrol	0.59±0.07^j-n^	428±28^b-h^	329±21^b-j^	49±0^l-o^	6.15±1.42^a-e^
Nk Technic	0.20±0.04^a-i^	442±5^b-i^	330±6^b-j^	39±1^e-m^	1.92±0.21^a-c^
Palmedor	0.20±0.03^a-i^	380±1^b-f^	307±4^b-i^	28±1^a-g^	4.09±0.99^a-e^
Pelican	0.37±0.06^c-m^	483±8^e-i^	373±10^e-k^	36±3^d-k^	2.94±0.86^a-d^
Pr45d01	0.07±0.01^a-d^	328±8^a-c^	255±10^a-d^	35±1^c-j^	3.67±1.23^a-e^
Pr45d03	0.04±0.01^a,b^	345±11^a-e^	236±4^a-c^	27±2^a-f^	4.00±1.88^a-e^
Pr46w10	0.02±0.01^a^	218±20^a^	183±19^a^	34±0^c-j^	Notdetermined
Pr46w31	0.11±0.03^a-f^	412±29^b-g^	313±23^b-i^	53±6^n,o^	2.00±0.43^a-c^
Shakira	0.75±0.10^n^	346±7^a-e^	298±6^b-h^	26±1^a-e^	1.21±0.18^a,b^
Taurus	0.45±0.07^h-n^	456±24^c-i^	400±26^g-k^	25±0^a-d^	3.20±1.08^a-d^
Toccata	0.17±0.04^a-h^	368±8^b-f^	290±6^a-g^	46±1^j-o^	3.50±0.92^a-e^
Totem	0.03±0.01^a,b^	432±16^b-h^	383±13^f-k^	25±2^a-d^	4.58±1.26^a-e^
Vectra	0.12±0.02^a-g^	425±31^b-h^	309±19^b-i^	30±2^a-h^	3.25±0.86^a-e^
Verona	0.20±0.06^a-i^	416±6^b-g^	331±12^b-j^	35±2^c-j^	1.21±0.13^a,b^
Viking	0.23±0.04^a-i^	373±8^b-f^	283±14^a-f^	40±2^f-m^	2.80±0.26^a-c^
Zeruca	0.06±0.02^a-c^	747±67^j^	607±66^l^	51±3^m-o^	9.15±2.45^e^
Zoom	0.30±0.10^a-j^	445±10^b-i^	419±12^i-k^	28±4^a-g^	1.63±0.23^a-c^

* *Samples composed of 40 flowers were collected, and nectar was harvested by centrifugation and quantified as described in Section Nectar Sampling. Results are mean ± SE over 12 replicates*.

§ *Sugar content was determined enzymatically, as described in Section Sugar Analysis*.

# *Total amino acid content was quantified by reaction with oPDA, as described in Section Amino Acid Analysis*.

*For a given parameter (nectar volume, glucose, fructose, sucrose, and total amino acid content), data were subjected to one-way ANOVA with post-hoc comparison using Tukey's honest significant difference test. In each column, means with the same letter are not significantly different (P > 0.05) from each other*.

**Figure 1:**
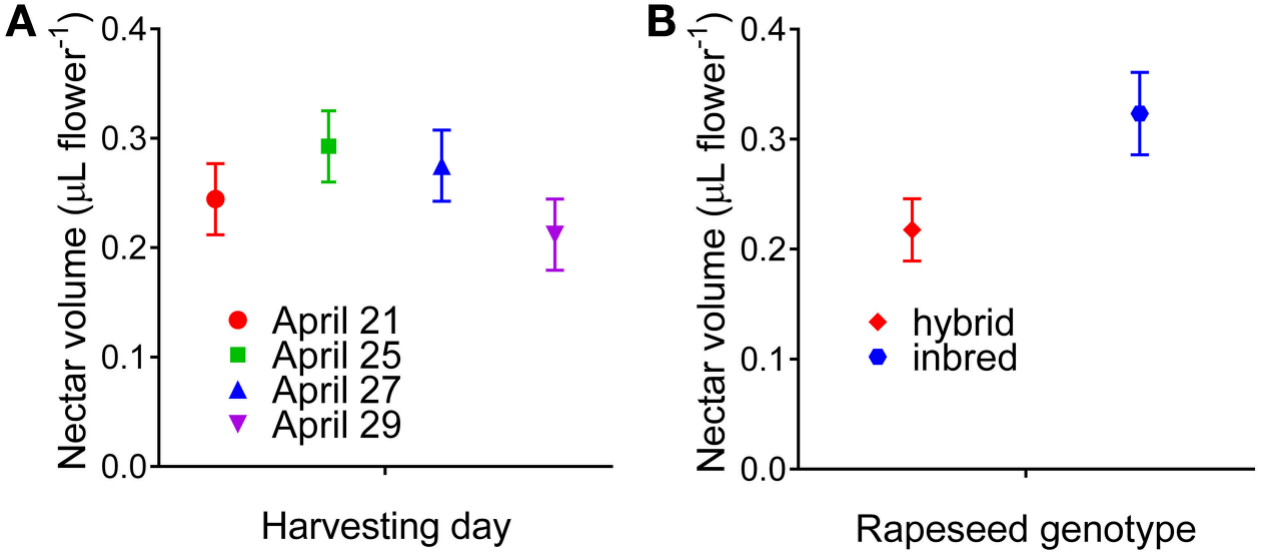
**Confidence intervals for mean nectar production**. Factorial ANOVA pointed out the occurrence of significant differences in nectar production by rapeseed cultivars also as a function of the day of harvest **(A)** and their genetic nature [hybrids vs. inbred lines **(B)**]. Reported intervals are at the 95% confidence level.

A lower but statistically relevant difference was also found with respect to the day of harvest (*P* = 0.003603). However, the interaction was not significant (*P* = 0.067888), suggesting that the variation of nectar production in different days was shared by all rapeseed genotypes, and that the relative ratio was not altered. The assessment of confidence intervals for mean nectar volumes in the 4 days of harvest (Figure 1A) pointed out maximal amounts on April 25^th^, in connection with increased values of relative humidity (Figure S3) due to a moderate rain that had occurred on April 23^rd^. Interestingly, when data were plotted as a function of the genetic background, hybrids showed a highly significant difference (*P* = 0.000012) from inbred lines. Confidence intervals for mean volumes (Figure 1B) showed that the latter produce about 50% more nectar than the former. In this case also, a minor yet significant difference (*P* = 0.028708) was evident with respect to the day of harvest, but again the interaction was not significant (*P* = 0.411980).

### Nectars from rapeseed genotypes show absolute concentrations of sugars that are notably different, but the percentage content of glucose, fructose, and sucrose is similar

**Figure 2:**
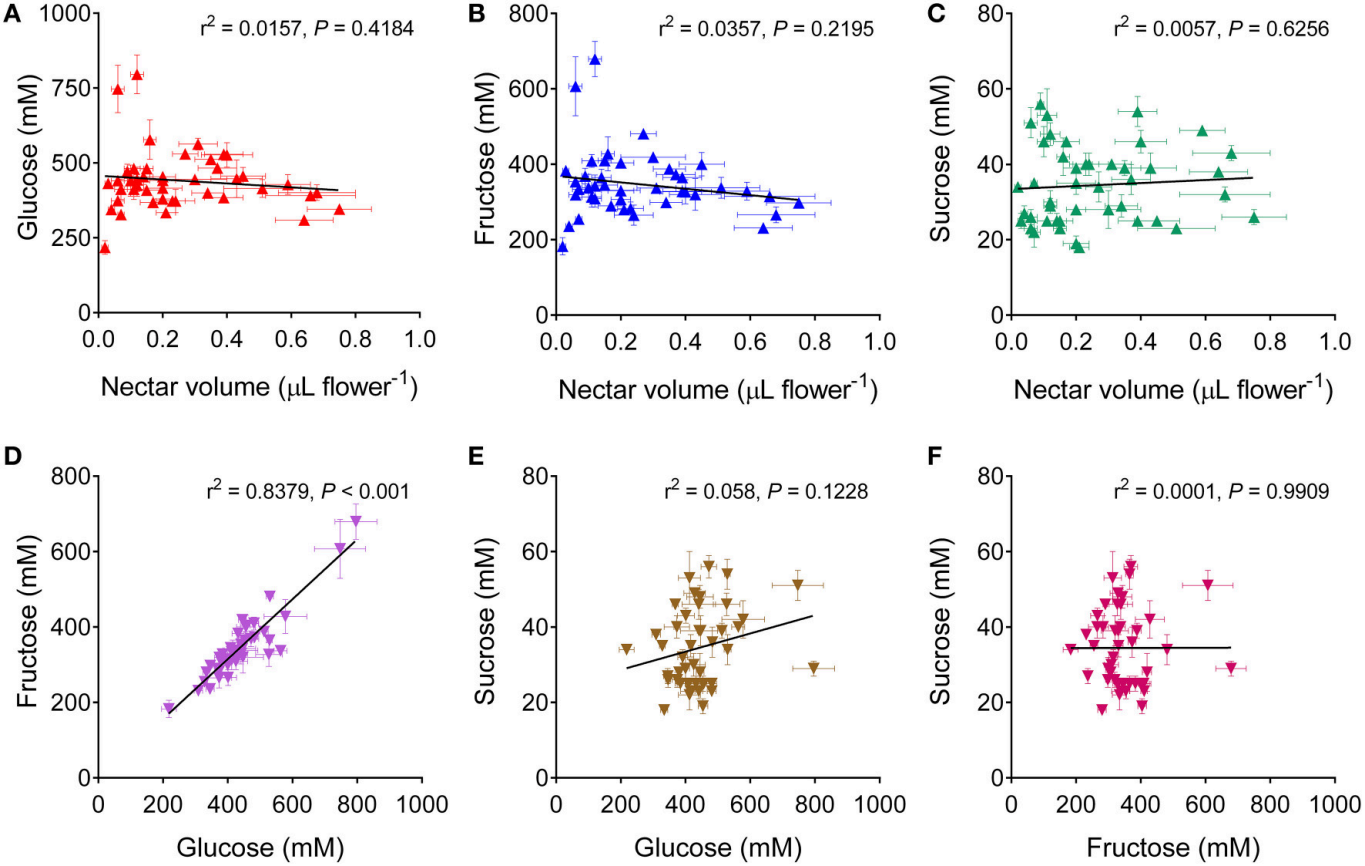
**Correlation analysis of sugar content in rapeseed nectar**. Glucose, fructose and sucrose concentrations in the nectar of a given cultivar were plotted against each other **(D–F)**, or against the corresponding nectar volume **(A–C)**. In all cases a two-tailed Pearson correlation test showed no significant relationship, with the only exception of glucose and fructose content, which were found to be highly correlated **(D)**.

The concentrations of glucose, fructose and sucrose in the harvested samples were measured on the basis of the calibration curves reported in Figure S4. Monosaccharides were nearly equally abundant, with concentrations ranging from 200 to 750 mM, whereas only minor levels of the disaccharides were detected, in the 20–60 mM range (Table 2). Genotypes differed significantly regarding the content of all three sugars, being *P* = 0.000019, *P* = 0.000001, and *P* = 0.000000 for glucose, fructose and sucrose, respectively. On the contrary, inbred lines and hybrids did not differ from each other with respect to the three sugars (*P* = 0.409379, *P* = 0.167797, and *P* = 0.647939), and the overall content (*P* = 0.321408). When sugar concentrations were related to the corresponding nectar volume, no significant relationship was evident for all the compounds (Figures 2A–C). This suggests that the differences found with respect to nectar volume do not depend only on a variable water content deriving from either sample contamination by dew (or other floral fluids), or on a variable dilution of a uniform exudate secreted by nectaries. On the contrary, a highly significant correlation was found between glucose and fructose content (Figure 2D), whereas sucrose concentration was related to neither of the monosaccharides (Figures 2E,F).

**Figure 3:**
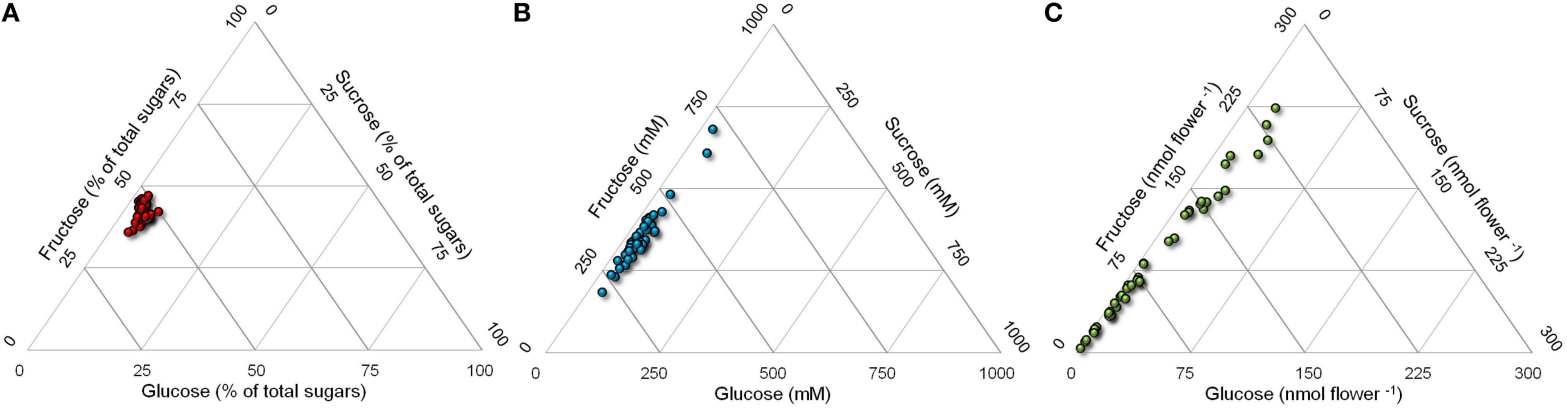
**Ternary plot of sugar content in rapeseed nectar**. Glucose, fructose, and sucrose levels in nectars from 44 rapeseed cultivars were plotted in triangular graphs depicting their relative ratios. Data are expressed as percentage content **(A)**, millimolar concentration **(B)**, or absolute amount *per* flower **(C)**.

Despite the interesting differences found with respect to absolute sugar concentrations (Figure 3B), if glucose, fructose and sucrose levels were expressed as percentage values, a more uniform content was evident, even though genotypes still differed statistically from each other (data not shown). Percentage content of glucose ranged from 48 to 62% total sugars, that of fructose from 33 to 48% and that of sucrose from 2 to 13% (Figure 3A), suggesting that the relative concentrations of these compounds are determined by mechanisms that are maintained in all genotypes. However, if the absolute content for each flower is considered, i.e. if for a given genotype the different levels of the three sugars are multiplied by the corresponding nectar volume, the variability among cultivars is even more pronounced, with glucose and fructose content ranging from 4 to 250–270 nmol flower^−1^ (Figure 3C). This implies that the reward for an insect foraging on a single flower differs dramatically among genotypes due to the absolute content of glucose, fructose and sucrose. Based on the data herein obtained, the caloric reward *per* flower would vary over a 50-fold range, from 0.03 joule in the case of the hybrid Pr46w10 to 1.50 joule for the inbred Shakira.

### Amino acid content in rapeseed nectars shows different absolute concentrations and relatively uniform percentage values

**Figure 4:**
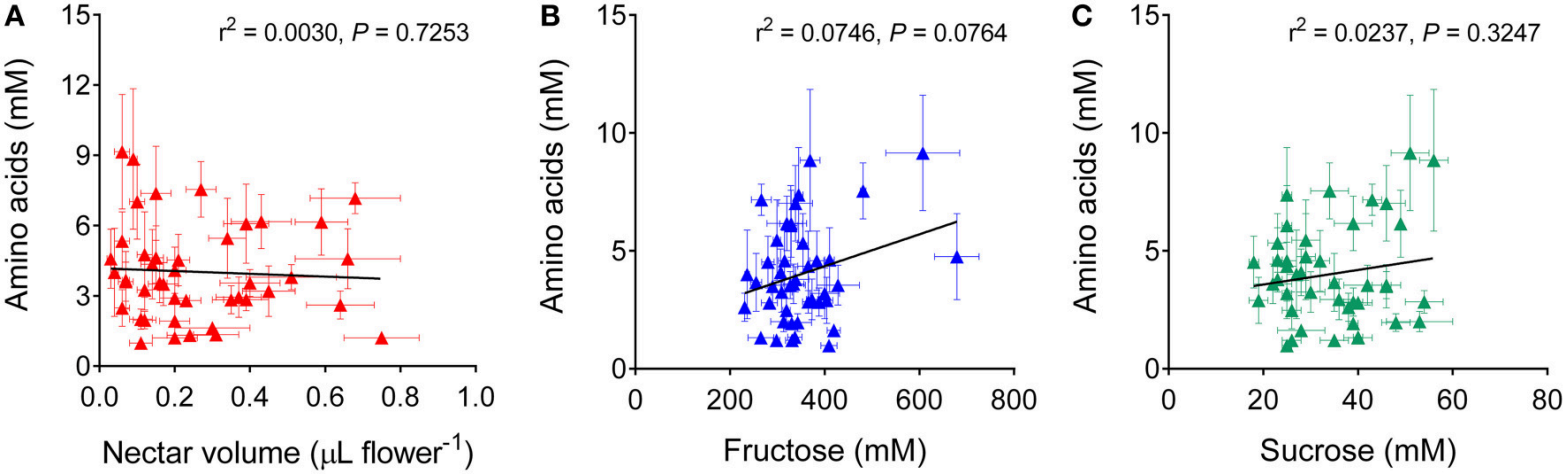
**Correlation analysis of amino acid content in rapeseed nectar**. Total amino acid concentration in the nectar of a given cultivar was plotted against the corresponding nectar volume **(A)**, or against fructose **(B)**, or sucrose **(C)** content. In all cases a two-tailed Pearson correlation test showed no significant relationship.

Total amino acid concentration was much lower than sugar levels, ranging from 1 to 9 mM. A remarkably high variability was found within cultivars (Table 2). With respect to this trait, rapeseed genotypes were significantly different (*P* < 0.0001). On the contrary, inbred lines and hybrids did not show a different amino acid content (*P* = 0.6110 in the unpaired *t*-test with Welch's correction). If amino acid concentrations were related to the corresponding nectar volumes, no significant relationship was found (Figure 4A), further strengthening the possibility that the variability pointed out with respect to the amount of nectar *per* flower reflects a true difference and does not depend on a different water content. Non-significant correlations were found also between amino acid levels and glucose (not shown), fructose (Figure 4B), or sucrose (Figure 4C) concentration.

**Figure 5:**
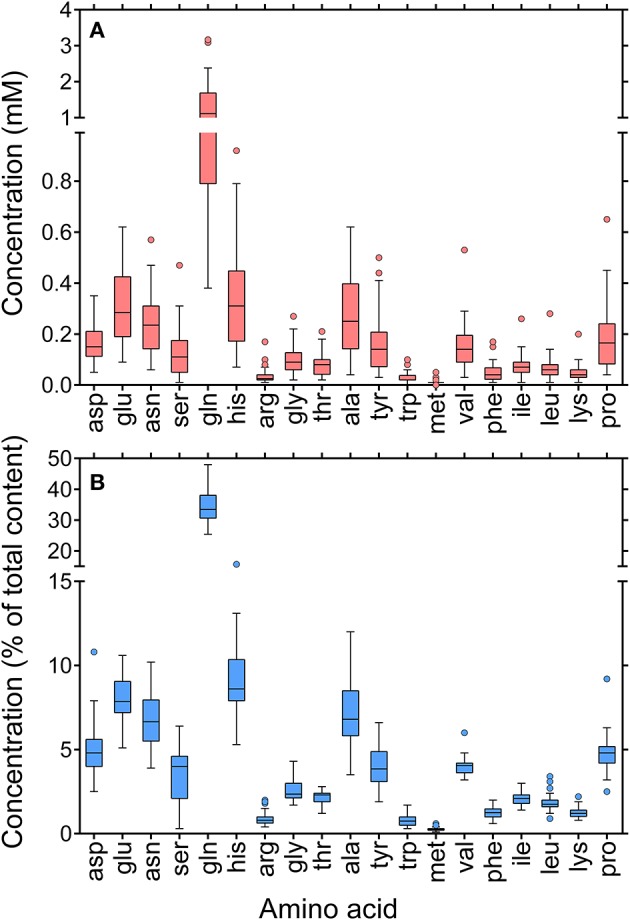
**Whisker-and-box plots of amino acid content in rapeseed nectar**. Single amino acid concentrations were determined by RP-HPLC analysis of a sample obtained by combining the same volume of all the 12 existing specimens for a given genotype. Because of the limited amounts of nectar available, only 36 of 44 cultivars were analyzed. Results were expressed as either absolute concentrations **(A)**, or percent values of total amino acid content **(B)**.

Because of the low concentrations and the limited availability of nectar, for most cultivars the analysis of individual amino acid content was carried out on a sample obtained by combining the same volume of all the 12 existing specimens. Results are shown in Figure 5. A striking variability was found concerning absolute concentrations (Figure 5A), reflecting the large variations in total amino acid content (Table 2). However, if data were plotted as percentage values, a much more uniform picture was obtained (Figure 5B). Similarly to the results found for sugar content, these data seem suggestive of shared mechanisms controlling the reciprocal ratios of free amino acids. In all cases glutamine was the predominant amino acid, accounting for about one third of total content. High percent values were also found for histidine, glutamate, asparagine, and alanine. Proline levels corresponded to around 5% of total amino acid content.

**Figure 6:**
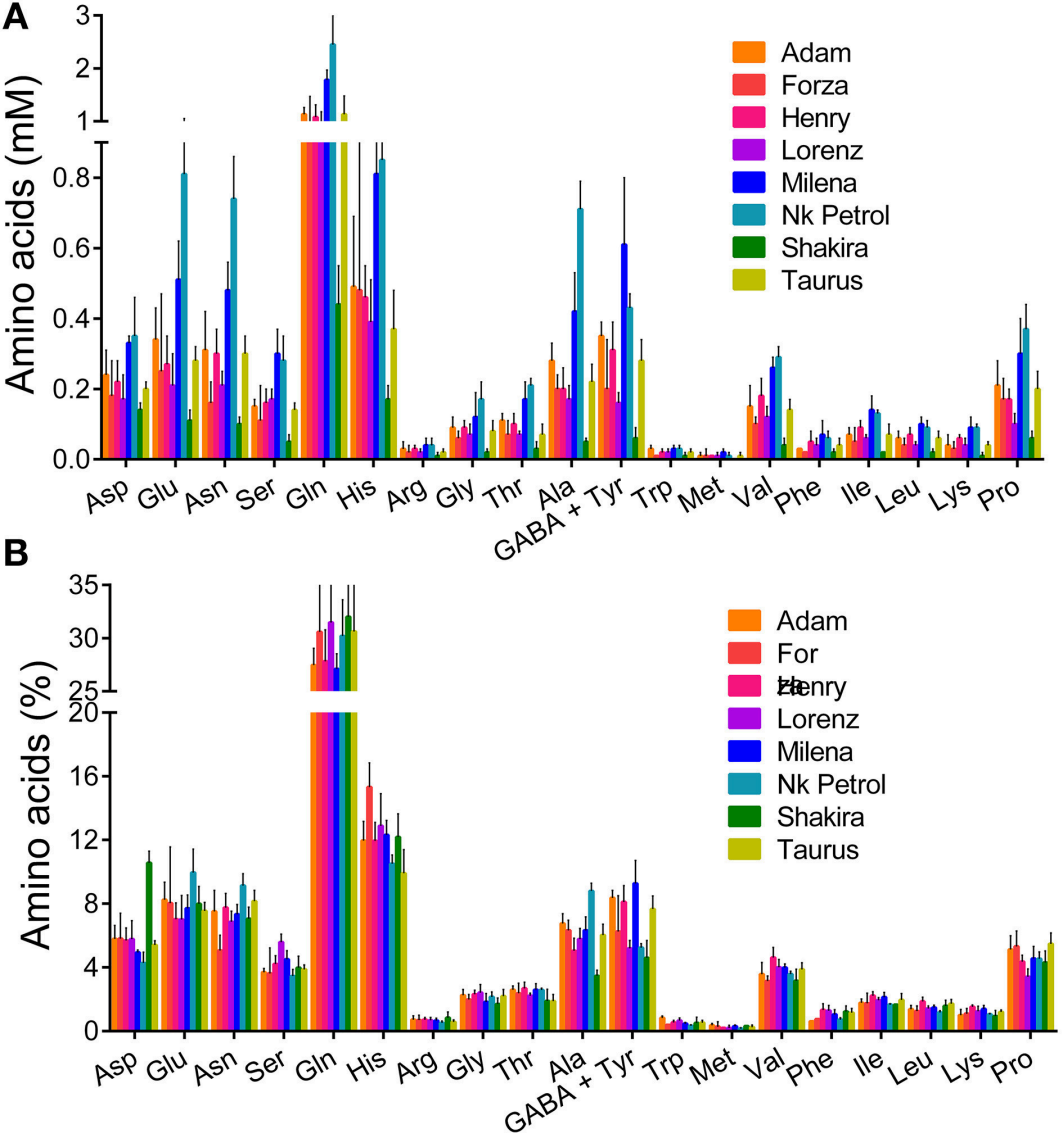
**Single amino acid content in a subset of eight rapeseed cultivars**. For those genotypes for which larger amounts of nectar were available, single amino acid concentrations were determined by RP-HPLC analysis of four samples obtained by combining the three specimens harvested in the same day. Results were expressed as either absolute concentrations **(A)**, or percent values of total amino acid content **(B)**.

For a subset of eight genotypes, the availability of larger nectar volumes allowed a suitably replicated analysis of individual amino acid content. Tabular numerical data are reported in Table S6, and results are summarized in Figure 6. Even within this small set of cultivars, absolute concentrations varied greatly (Figure 6A), and showed a significant variability (*P* = 0.0025). On the contrary, percentage data (Figure 6B) did not differ significantly among genotypes (*P* > 0.9999).

### Sugar and amino acid content of phloem sap from a subset of eight rapeseed cultivars shows a remarkable uniformity, and both absolute and relative values are completely different from those found in nectar

Since floral nectar derives from phloem sap, the composition of the latter was investigated in the case of the small group of genotypes for which a complete analysis of sugar and amino acid content of nectar had been obtained. Data on sucrose, glucose, fructose, and total amino acid concentrations in phloem sap are presented in Table 3. As expected, sucrose was the main saccharide, accounting for more than 80% of total sugars, whereas monosaccharides were present at a tenfold lower concentration. Interestingly, free amino acid content was similarly high, ranging from 180 to 250 mM.

**Table 3:** **Sugar and amino acid content of rapeseed phloem sap**.

**Cultivar**	**Sucrose (mM)**	**Glucose (mM)**	**Fructose (mM)**	**Amino acids (mM)**
Adam	442±9^a^	46.7±15.3^a^	38.8±11.6^a^	230±19^a^
Forza	420±31^a^	26.9±5.3^a^	22.4±4.7^a^	199±15^a^
Henry	493±74^a^	30.7±5.4^a^	25.5±4.7^a^	226±20^a^
Lorenz	440±25^a^	35.9±3.8^a^	31.3±4.0^a^	195±16^a^
Milena	476±32^a^	30.4±2.8^a^	24.9±2.3^a^	224±16^a^
Nk Petrol	446±19^a^	27.7±2.1^a^	24.3±1.4^a^	219±28^a^
Shakira	494±41^a^	47.5±11.0^a^	39.7±8.9^a^	249±26^a^
Taurus	397±27^a^	31.2±4.8^a^	27.4±4.4^a^	187±11^a^

*For a given parameter (sucrose, glucose, fructose, and total amino acid content), data were subjected to one-way ANOVA with post-hoc comparison using Tukey's honest significant difference test. In each column, means with the same letter are not significantly different (P > 0.05) from each other*.

**Figure 7:**
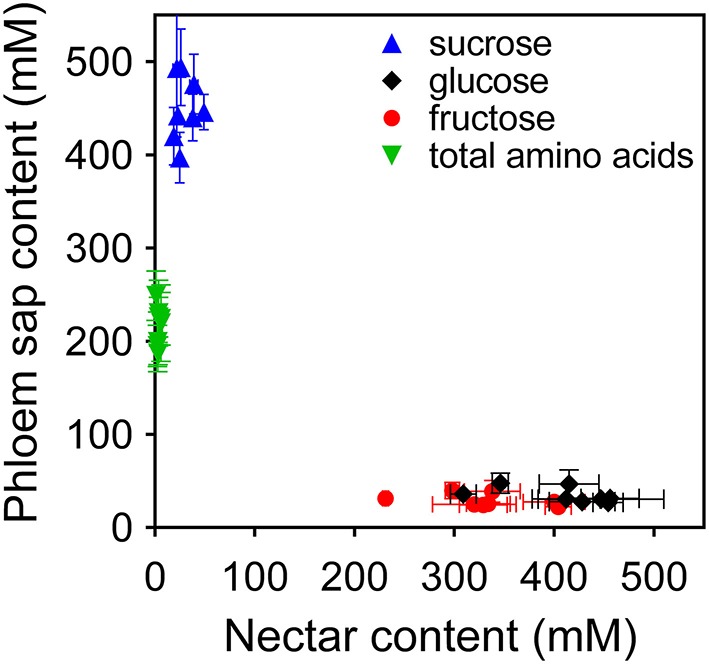
**Comparison of sugar and amino acid content in rapeseed nectar and phloem sap**. The concentrations of glucose, fructose, sucrose, and total amino acid found in nectar were plotted against those found in phloem sap. Data, expressed as mean values ± SE, refer to the subset of eight genotypes for which sap had been analyzed.

When sugar and amino acid content in phloem sap of a given cultivar was related to the corresponding concentration in nectar, significant differences were evident (Figure 7). If absolute sugar concentrations were considered, data are suggestive of a major hydrolysis of sucrose in its two components by nectaries, the overall content being comparable (700–1000 mM, as expressed in monosaccharide equivalents). On the contrary, high amino acid concentrations in sap were strongly reduced in nectar. Moreover, unlike nectar, both sugar and amino acid concentrations in sap samples were remarkably similar in all tested varieties, the differences being statistically non-significant (*P* = 0.3394 for sugars in one-way ANOVA for matched measures, and *P* = 0.1494 including also amino acid content).

**Figure 8:**
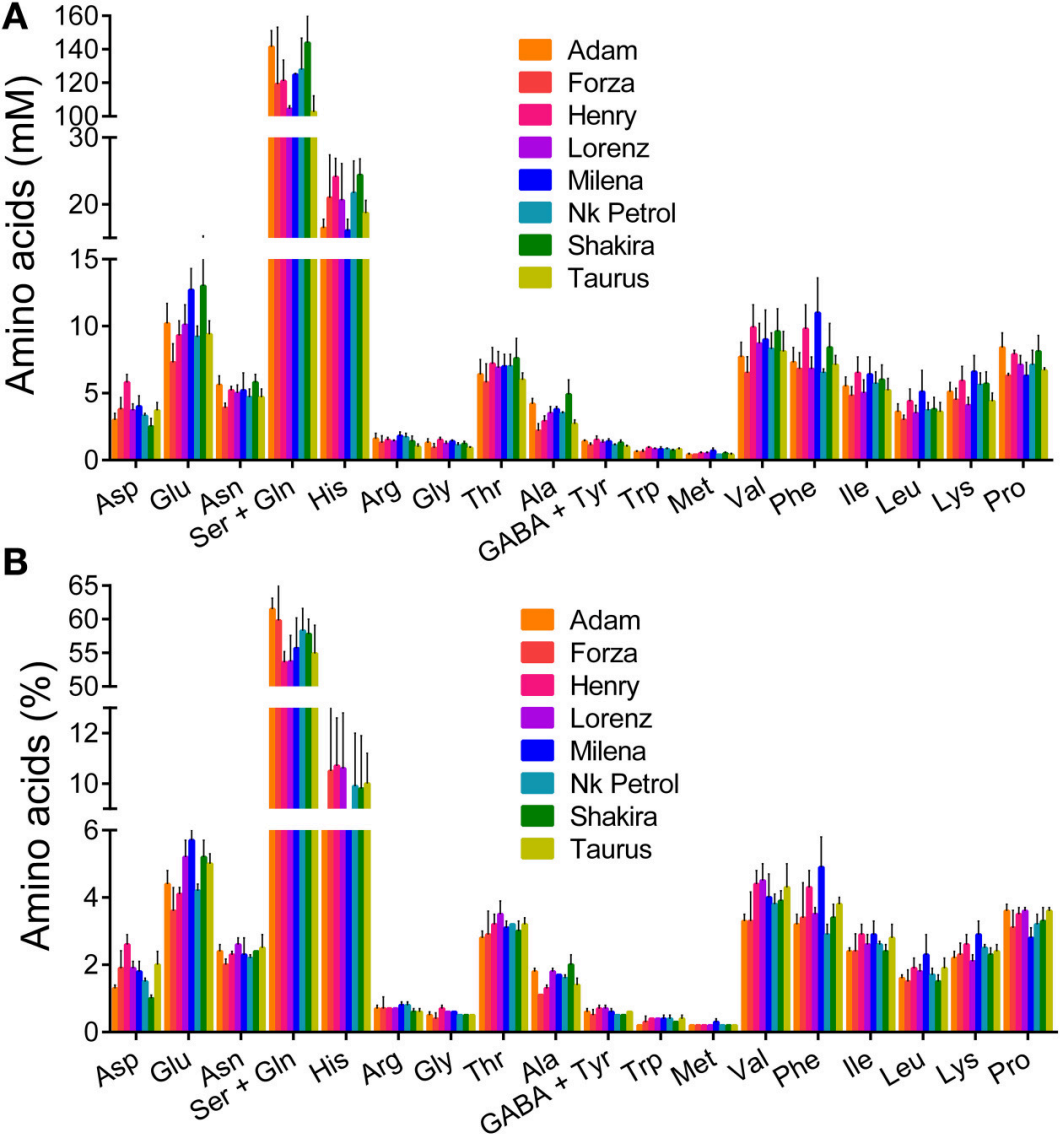
**Single amino acid content in phloem sap**. For a subset of eight genotypes, single amino acid concentrations in phloem sap were determined by RP-HPLC analysis. Results are means ± SE over three biological replications, and were expressed as either absolute concentrations **(A)**, or percent values of total amino acid content **(B)**.

**Figure 9:**
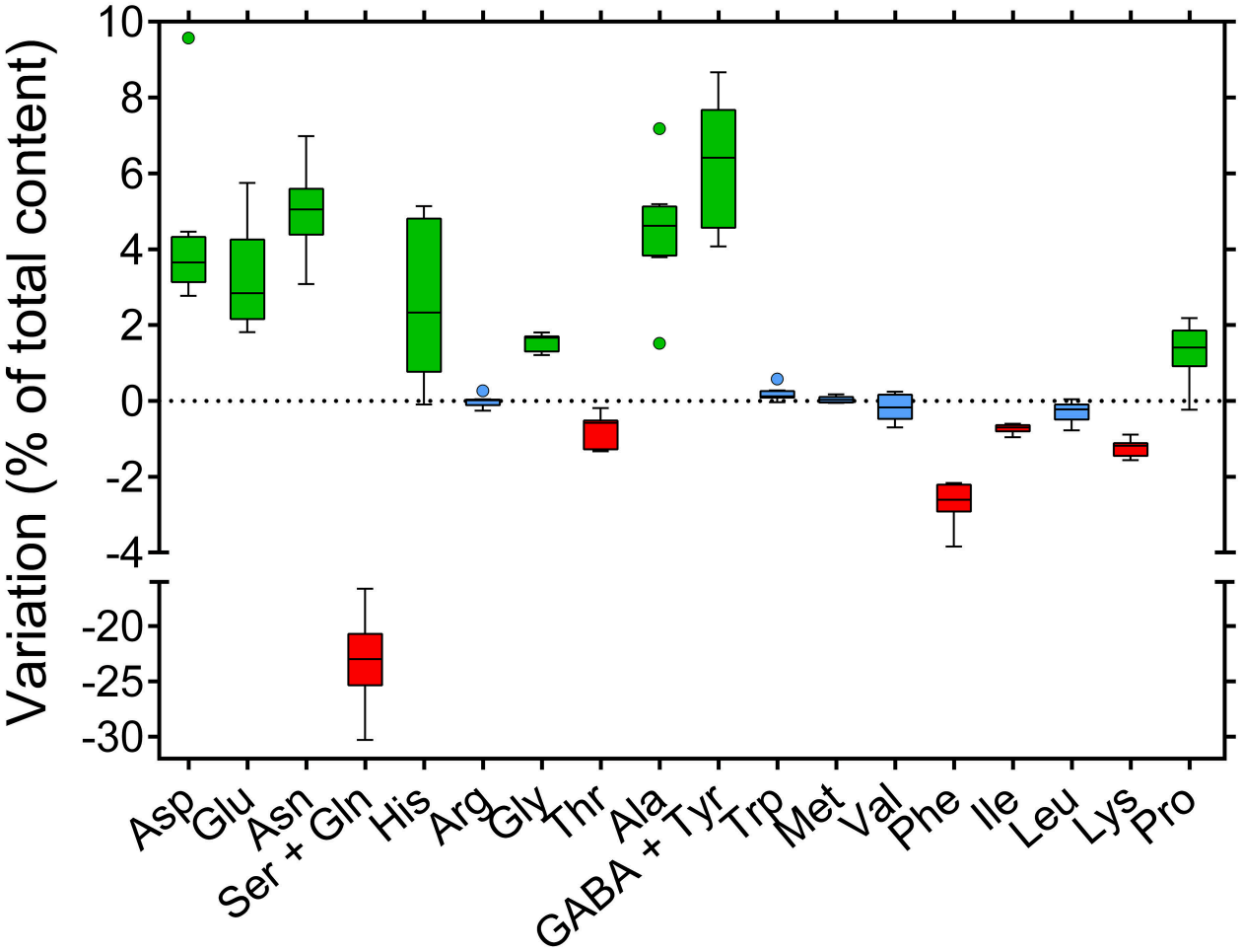
**Variation of single amino acid content between nectar and phloem sap**. The levels of free amino acids found in nectar were compared with those found in phloem sap. For each compound data were expressed as the variation of its percent contribution to total amino acids. Whisker-and-box plots obtained for the subset of eight genotypes whose phloem sap had been analyzed are presented.

The last result was further confirmed when single amino acid levels were determined in phloem sap (Table S7). Contrary to previous results for nectar (Figure 6), and despite a much lower intra-genotype variability, differences among rapeseed varieties were not significant either when expressing single amino acid content as absolute concentrations (Figure 8A, *P* = 0.1932), or when plotting data as percentage values (Figure 8B, *P* = 0.9969). Considering variations between nectar and phloem sap, absolute differences were not very informative, nectar content being strongly reduced in all cases. If expressed as percentage differences, data showed that some amino acids were proportionally enriched in nectars, whereas others were reduced (Figure 9). Among the latter the main variation concerned glutamine, whose contribution to total amino acids halved. Proline content increased slightly, similarly to several other amino acids, among which aspartic and glutamic acid, asparagine, and γ-aminobutyric acid.

## Discussion

This study aimed at investigating the occurrence of intraspecific variability among cultivated rapeseed winter genotypes with respect to sugar and amino acid content in nectar. Sugar concentration and quality can significantly influence both foraging insect preference and, in the case of bees, the properties of the resulting honey. For instance, a high glucose-to-fructose ratio can cause increased tendency of honey to granulate even in the hives, forcing beekeepers to adopt special process of harvesting. Moreover, honeybee preference may positively affect also seed set efficiency, possibly leading to significant increase of crop yield (Abrol and Shankar, 2012). Several previous reports on rapeseed nectar showed no or very low sucrose concentrations, and a relatively low fructose-to-glucose ratio, in the range 0.80–0.95 (Westcott and Nelson, 2001). These data were generally obtained on a small number of rapeseed cultivars (e.g., Mohr and Jay, 1990). Only a very few studies to date have investigated nectar properties in a wide array of rapeseed genotypes (Kevan et al., 1991; Pierre et al., 1999). In those surveys only limited characterization was performed. In the former study, only glucose and fructose concentrations in nectars of 25 cultivars were considered (Kevan et al., 1991). In the latter case, the variability in nectar secretion among 71 genotypes of winter oilseed rapes was tested for floral nectar volume, sugar composition, and concentration (Pierre et al., 1999). In both cases, neither amino acid level, nor the relationship between sugar and amino acid content of nectar and phloem sap was determined.

The present results highlighted the occurrence of a remarkable variability among rapeseed genotypes concerning nectar volume, with mean values ranging from 0.02 to 0.75 μL flower^−1^. A lower, yet significant eight-fold variation had also been reported in one of the aforementioned studies (Pierre et al., 1999). In that case, however, much higher absolute values had been found, from 0.7 to 5.9 μL flower^−1^. Such a discrepancy may depend on the adoption of a different protocol for nectar harvesting (centrifugation vs. collection with micropipettes) or, more probably, on different field conditions. In fact, in that as well as in most other previous works (e.g., Mesquida et al., 1988; Nedić et al., 2013) nectars were sampled from flowers bagged 24 h prior to sampling in order to prevent nectar uptake by foraging insects. On the contrary in the present study freshly-opened, unvisited flowers were harvested from plants that were continuously visited by bees and other foraging insects, which is a much more natural condition. It is quite likely that in completely unvisited plants nectar could accumulate with time, reaching higher volumes *per* flower.

An increasing amount of data emphasized that nectar volume and composition may be significantly influenced by a number of factors, including relative humidity, time of day, and soil composition. For instance, the flowers of some cultivars of both *B. napus* and *B. campestris* produced more nectar -with a lower sugar content- in the morning, and correlations were found between the amounts and concentrations of nectar produced and temperature or relative humidity (Mohr and Jay, 1990). In the present study, special attention was paid to standardize nectar harvesting conditions, as to obtain reproducible results and allow a proper comparison among rapeseed genotypes. Nectar collection was not carried out in the morning, when the relative humidity is higher and samples may be contamined by dew, but in the early to mid-afternoon, when the establishment of full photosynthetic rate and phloem loading should lead to the attainment of steady-state nectar production. Moreover, harvesting was performed in sunny days, with similar temperature and humidity (Figure S3). This notwithstanding, significant variations were found between nectar volumes harvested in different days (Figure 1A). Consistently with literature data (Mohr and Jay, 1990), the highest values were obtained following a moderate rain that occurred after the first harvesting day, despite the fact that the second harvest had been postponed for this reason. However, lowest and highest mean values differed by < 40%, whereas in other studies up to three-fold variations were reported (Pierre et al., 1999). Moreover, the results of a factorial analysis of variance showed that the increase was not dissimilar in all genotypes, strengthening the conclusion that the differences found reflect true differences in nectar production.

Besides environmental conditions, nectar content may also vary depending on floral sexual phases (Antoñ and Denisow, 2014) and flower position within inflorescences (Lu et al., 2015). However, these aspects were not considered, the aim of this study being to compare nectar from different genotypes. The use of highly standardized and uniform harvesting conditions should have minimized the effect of these variability factors. Interestingly, besides the highly significant difference among genotypes, nectar volumes from rapeseed inbred lines were found to differ significantly from those produced by hybrids, with the mean value of the latter about 50% lower than the former (Figure 1B). Such a difference might depend at least in part on the presence of a higher number of flowers *per* plant in hybrids, the number of pods being one of the eight yield-correlated traits showing significant mid-parent heterosis found in rapeseed (Shi et al., 2011). To the best of our knowledge, no other information is available to date regarding this point, since in the previous survey in which a significant number of rapeseed genotypes were considered, only one hybrid was included among 71 rape lines. However, in that case a significant difference with respect to nectar volume was found among three types of conventional fertile oilseed rape lines and varieties differing in seed quality, i.e. containing low (0) or high (+) levels of erucic acid and glucosinolates (00, 0+, and ++, respectively; Pierre et al., 1999). Whatever the reason for such a difference, this could result in a lower preference of honeybees for hybrid varieties, the caloric reward *per* flower being significantly reduced. If so, a lower impollination rate could partially counteract the outbreeding enhancement.

With respect to sugar content, the results herein described are on the whole consistent with those of previous reports, in all cases glucose concentration being higher than that of fructose, and sucrose concentration much lower than both of the two hexoses. However, significant levels of sucrose were found in all samples and, if expressed as percent of total sugar content, they were remarkably higher than previously reported, ranging from 3.7 to 14.3%, with a mean value of 8.2%. As a basis for comparison, Pierre et al. (1999) reported a mean sucrose content ranging from 0.3 to 0.6%. This discrepancy may depend on the different analytical techniques used (enzymatic assays vs. HPLC quantitation). However, it cannot be excluded that also in this case different results may derive from different conditions under which nectar sampling was carried out. In flowers bagged for 24 h before harvesting, nectar might be processed for a longer time by nectaries, allowing an almost complete hydrolysis of sucrose. On the contrary, in plants continuously visited by foraging bees nectar could be produced at higher speed to replace the amounts taken by insects, without the time to complete the invertase reaction. Further work will be required to discriminate between these hypotheses. Concerning absolute sugar concentrations, highly significant differences were found among rapeseed genotypes, ranging from 0.468 to 1.532 M hexose equivalents. In a previous study no clear genotypic effect on sugar levels could be demonstrated. However, in that case the analysis was carried out to verify divergence among varieties differing in seed quality, and genotypes of each type were not equally represented on every harvesting date (Pierre et al., 1999). Therefore, this is the first report clearly showing the occurrence of a high variability in rapeseed regarding this trait, as shown to occur in other Brassicaceous species (Denisow et al., 2015).

This is also the first report describing free amino acid content in rapeseed nectar. With respect to both total amino acid content and single amino acid composition, the genotypes analyzed showed a high degree of diversity. Consistently with literature data on other species (e.g., Gardener and Gillman, 2001a; Carter et al., 2006), the overall absolute concentrations ranged from 1 to 10 mM. Glutamine was the most abundant, accounting for about one third of total content. Relatively high levels were also found for histidine, glutamate, asparagine, and alanine. Such a composition is quite different from the ideal ratio of essential amino acids that are required for the normal growth and development of bees (De Groot, 1953). Moreover, the content of the only amino acid that most insects have the ability to taste, proline (Wacht et al., 2000; Gardener and Gillman, 2002), was quite low in all genotypes, and below the levels (1–5 mM) that were found in nectars from other plant species (Carter et al., 2006; Nepi et al., 2012) and preferred by bees in dual choice feeding experiments (Carter et al., 2006; Bertazzini et al., 2010). On the whole, it therefore seems that amino acid profile in rapeseed nectars differs from that hypothesized to be attractive for foraging insects. In fact, it is believed that rapeseed foraging honeybees would obtain essential amino acids and protein mainly from the large amount of pollen available from rapeseed, which shows a nutritionally balanced amino acid composition (Somerville, 2001). Moreover, recent data seem to point to a much more complex picture for the relationship between amino acid content and bee preference. For instance, the honeybee's nutritional state was found to influence the likelihood it would feed on and learn sucrose solutions containing single amino acids (Simcock et al., 2014). Moreover, the nutritional balance of essential amino acids and carbohydrates of the adult worker honeybee was shown to depend on age, and foragers were found to require a diet high in carbohydrates (essential amino acids:carbohydrates 1:250), and showed low survival rates on diets high in amino acids (Paoli et al., 2014).

Whatever the effects on insect foraging preference, the present results showed a significant intraspecific variability with respect to both sugar and amino acid content in rapeseed nectars. This is consistent with an increasing amount of evidence (Lanza et al., 1995; Wolf et al., 1999; Leiss et al., 2004; Herrera et al., 2006) that superseded an early view of a substantial constancy of nectar composition (Baker and Baker, 1977). Several environmental factors have been shown to influence nectar production. For instance, nitrogen fertilization was found to greatly influence amino acid content (Gardener and Gillman, 2001b; Gijbels et al., 2015). However, due to the adoption of uniform conditions for growth including sufficient nitrogen supply, the variability found in this study could be genetic. The characterization of phloem sap content in a subset of rapeseed genotypes shed some light on this aspect. Despite the reduced number of cultivar considered, the comparison between phloem sap and nectar content clearly showed that the latter depends on the metabolic activity of nectaries. Concerning sugars, sucrose in phloem sap is hydrolyzed into its components. If expressed as hexose equivalents, concentrations in nectar and phloem sap are quite similar. However, the glucose:fructose ratio deviates significantly from the expected 1:1, in rapeseed as well as in many other species. This discrepancy strengthens even more the role of nectaries in determining the final composition of nectar. After the hydrolysis of sucrose, the hexoses are partially cycled through various biochemical pathways before being secreted into the lumen of the nectary, and this complex metabolism could explain the different ratios observed (Brandenburg et al., 2009). With respect to free amino acids, concentrations in phloem sap and nectar show, on the contrary, a striking divergence in rapeseed, the levels in the latter being 5% of the former. Absolute concentrations of individual amino acids are much more variable than their relative ratios. In phloem sap, glutamine accounts for two thirds of total amino acid content (Figure 8), most likely serving as the main form for organic nitrogen transport because of a high N:C ratio. In nectar, its percent contribution halves (Figure 6), concomitantly with a general reduction of amino acid content. It seems likely that most amino acids are retained by nectaries to sustain their active metabolism. Most interestingly, both sugar and amino acid composition of phloem sap showed a significantly higher uniformity than that of nectar, pointing to a low variability of the former among genotypes (Table 3, Figure 8). Therefore, the variability found in nectar seems to rely mainly on a different metabolization of a relatively constant phloem sap by nectary cells.

Although floral nectar traits are important for plant reproduction, little is known about their genetic basis. Only a few studies have quantified heritable variation for nectar traits (Mitchell, 2004). Our study suggests that nectary-specific expression levels of selected enzymes may play a main role in determining the variability found among rapeseed genotypes with respect to sugar and amino acid composition of nectar. Concerning nectar volume, some experimental evidence showed an essential role of jasmonic acid levels, integrating the floral nectar secretion into the complex network of oxylipine-mediated developmental processes of plants (Radhika et al., 2010). Future work will be required to verify whether the resulting variability corresponds to a different degree of preference by foraging insects. Irrespective of this aspect, the existence of an intraspecific variability implies the possibility of breeding for the attainment of increased concentration of selected nectar components. Moreover, the identification of nectary-specific promoters in an increasing number of species and some advances in the functional genomics of nectar production (Bender et al., 2012) are opening the way toward the tailoring of nectar composition through genetic transformation.

## Author contributions

MB performed most of the experiments; GF conceived and planned the research, performed a part of the experimental work, analyzed the results and wrote the paper.

### Conflict of interest statement

The authors declare that the research was conducted in the absence of any commercial or financial relationships that could be construed as a potential conflict of interest.
