# Supplementary material for: Intraspecific Variability of Floral Nectar Volume and Composition in Rapeseed (Brassica napus L. var. oleifera)
Source: Front Plant Sci. 2016 Mar 16;7:288. doi: 10.3389/fpls.2016.00288 (PMC4792878; doi:10.3389/fpls.2016.00288)
Supplement: Supplementary file 1 [file DataSheet1.pdf]

Table S1 │ Mechanical and chemical analysis of soil at the experimental field

Property	Method	
Particle size distribution (g/100 g)	Bouyoucos	
sand (2-0.02 mm)		35
silt (20-2 µm)		40
clay (< 2 µm)		25
texture (USDA classification)		loam
pH (1:2.5)		7.4
conducibility (mS cm^{-1}) (1:5)		0.61
Organic carbon (%)	Walkley Black	1.2
Organic matter (%)	Walkley Black	2.1
Calcium carbonate (%)	gas volumetric	8.0
Total nitrogen (%)	Kjeldhal	0.09
Available nitrogen (mg/kg soil)	potassium chloride	38.3
C/N ratio		13.4
Available phosphorus (mg/kg soil)	Olsen	10.0
Available magnesium (mg/kg soil)	ammonium acetate	144.4
Available potassium (mg/kg soil)	ammonium acetate	126.0
Cation-exchange capacity (meq/100 g)	Tucker	14.2

The field was divided into uniform soil areas; eight soil samples to 40 cm depth were collected at random over the field and mixed to give a composite sample.

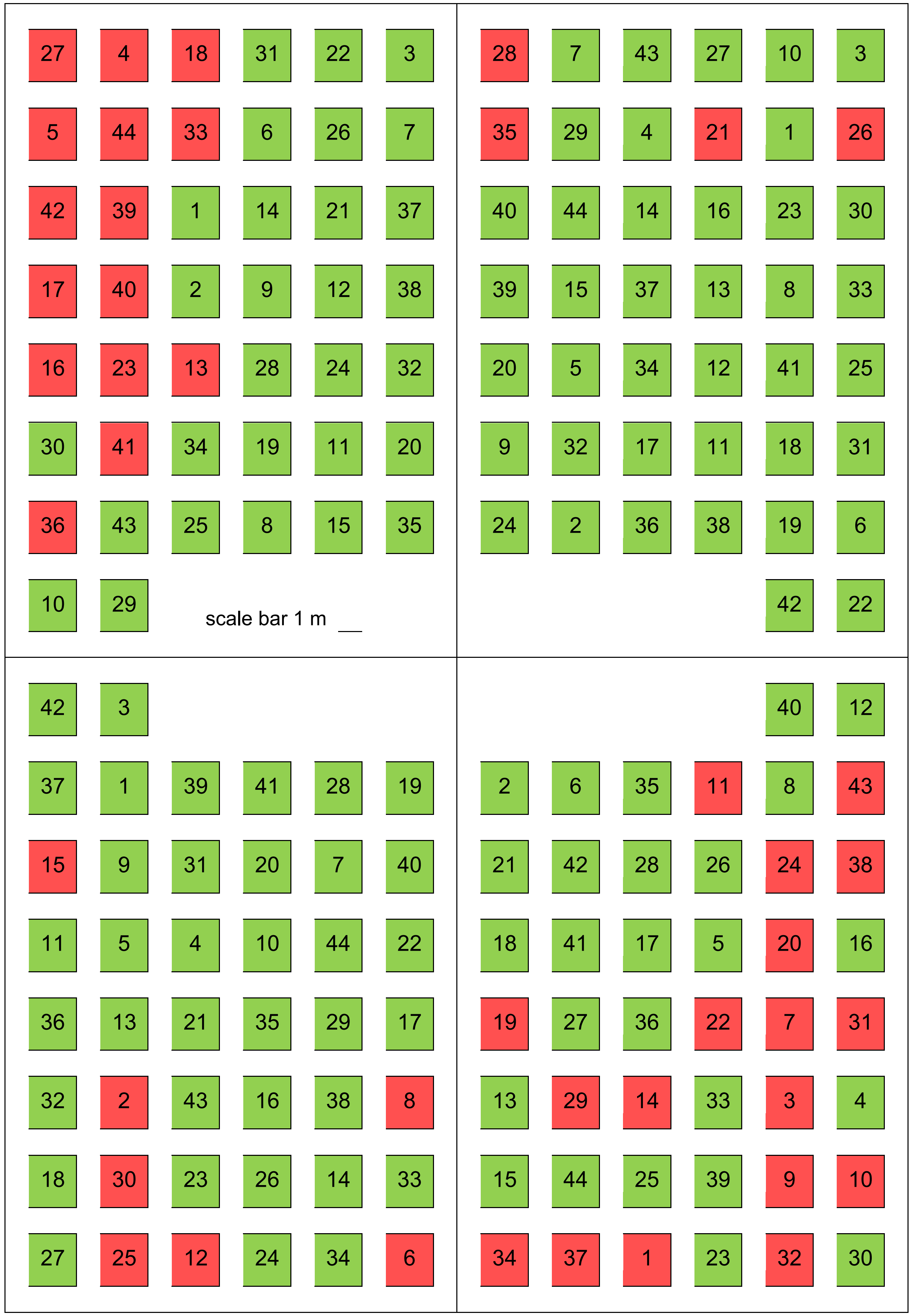

1 Hybristar
2 Forza
3 Dante
4 Vectra
5 Hercules
6 Milena
7 Pr45d03
8 Excel
9 Exagone
10 Excalibur
11 Gamin
12 Toccata
13 Es Artist
14 Katabatic
15 Adam
16 Kompass
17 Shakira
18 Henry
19 Verona
20 Nk Caravel
21 Nk Technic
22 Nk Petrol
23 Nk Formula
24 Pr45d01
25 Pr46w10
26 Pr46w31
27 Viking
28 Elan
29 Lorenz
30 Aragon
31 Taurus
32 Alpaga
33 Zoom
34 Totem
35 Beluga
36 Facile
37 Intense
38 Bambin
39 Avenir
40 Belcanto
41 Pelican
42 Zeruca
43 Palmedor
44 Mendel

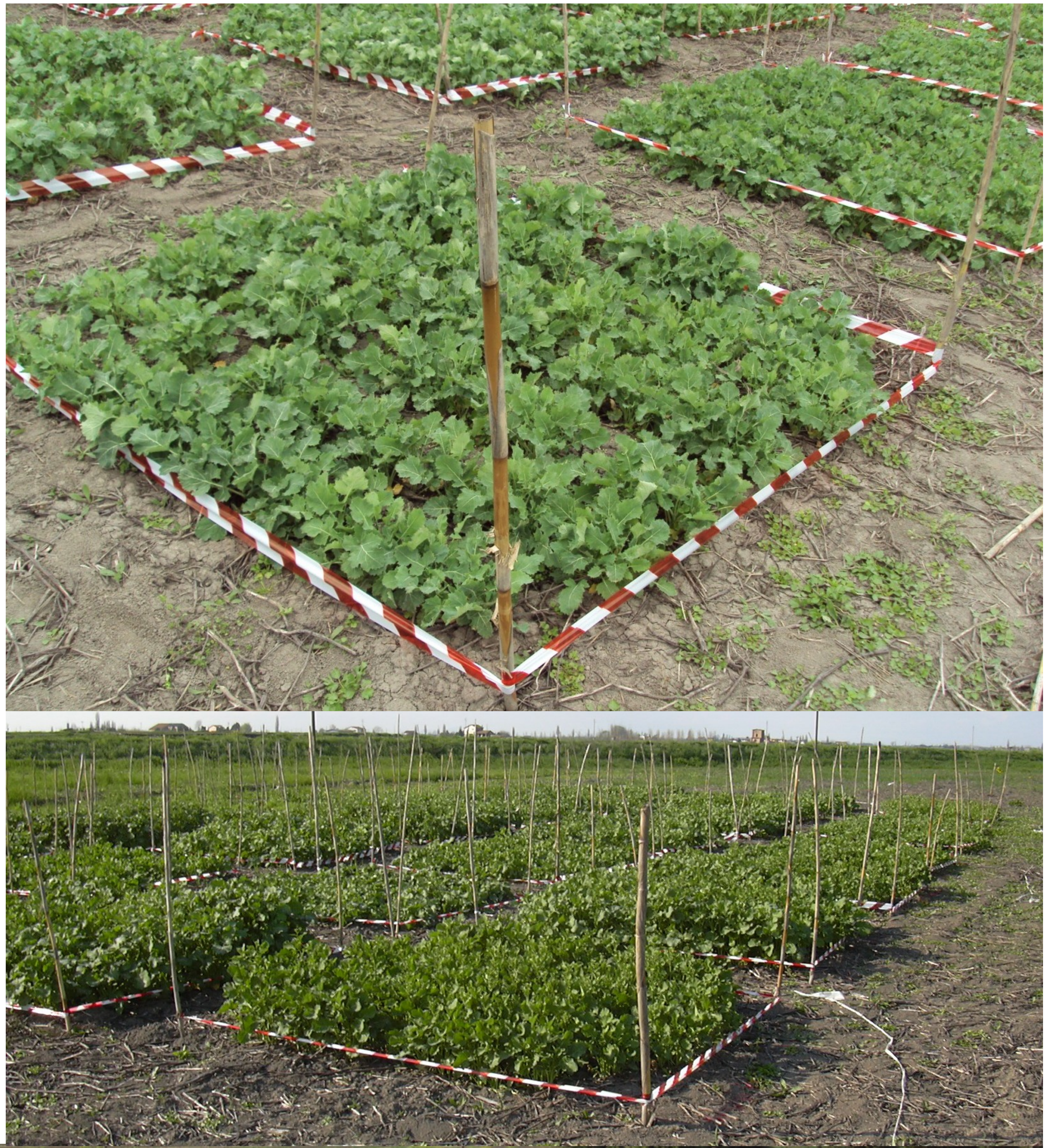

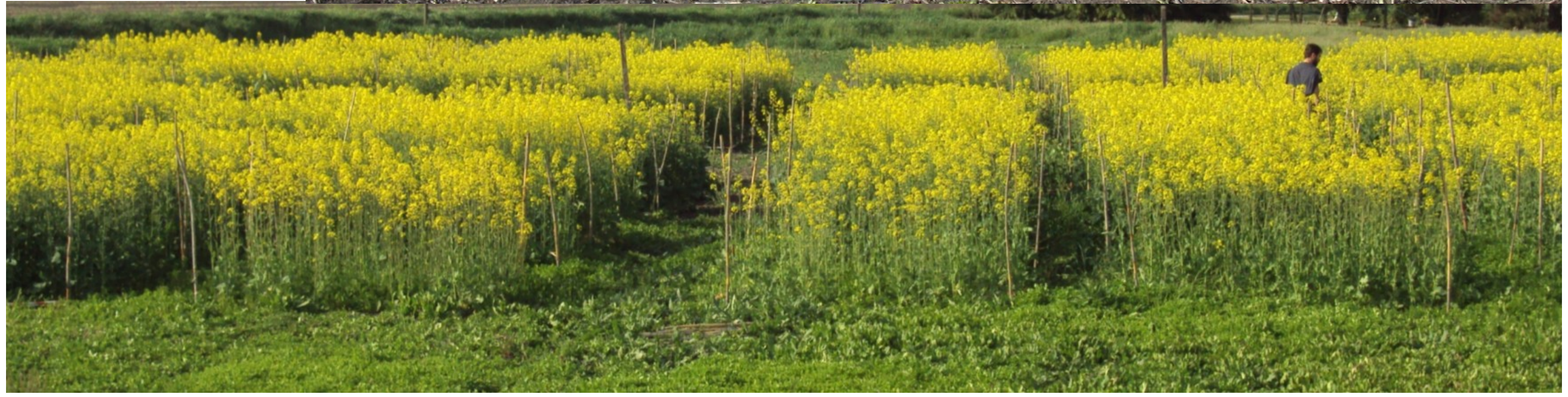

Figure S2 │ The randomized experimental design used in this study. The field was divided into four parcels. Each parcel enclosed 44 plots (2 x 2 m), each one consisting of seven rows, 33 cm apart. Just before nectar sampling, three plots with plants showing a uniform growth were selected for each rapeseed variety (in green), whereas plants in the fourth plot (in red) were not used. Nectars were harvested from plants at the 63 to 65 phenological stage in the BBCH-scale (as shown in the photograph at the right bottom).

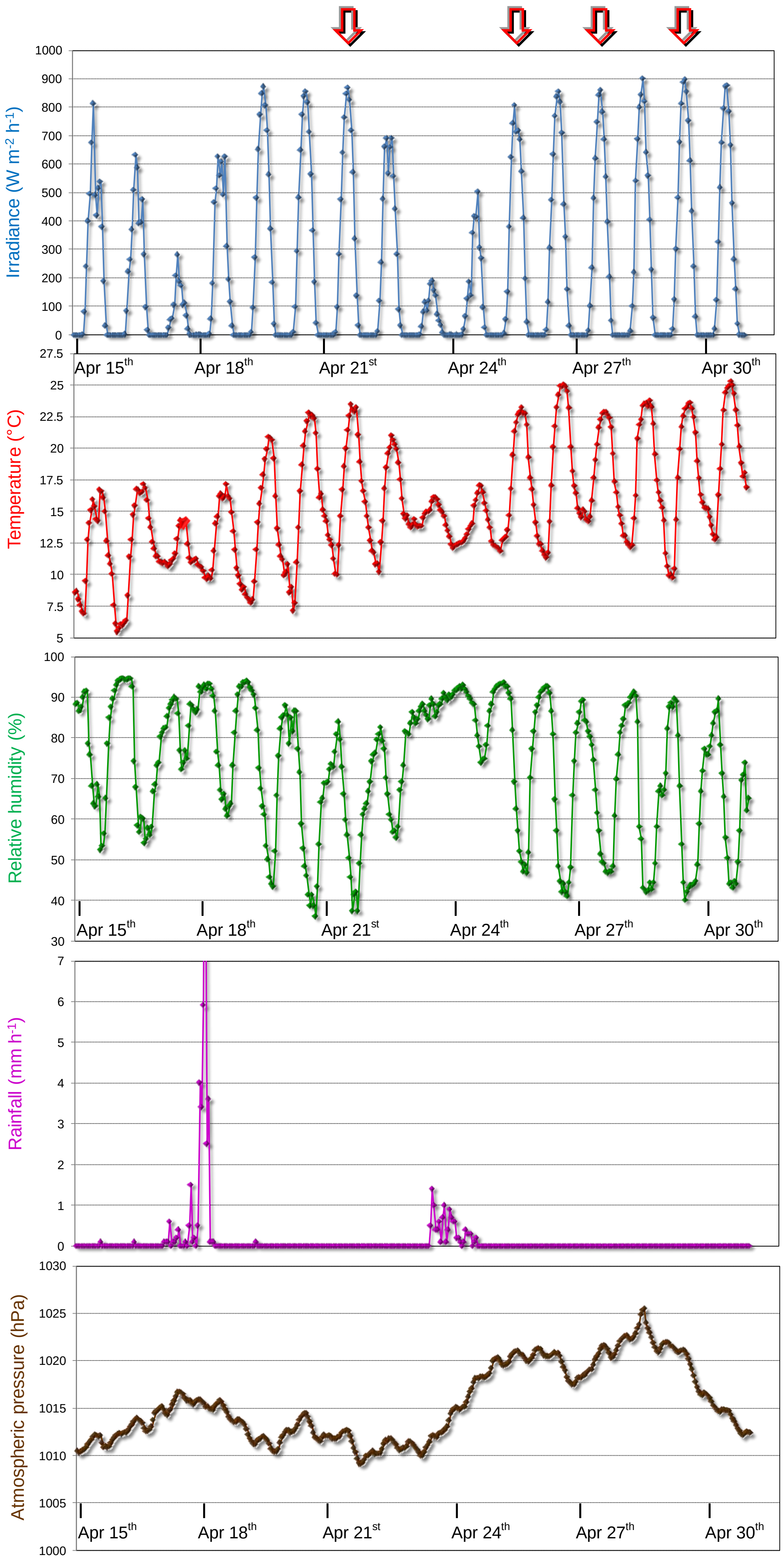

Figure S3 │ Atmospheric conditions immediately before and during nectar harvesting (⇩). Data were retrieved from the *Dexter* database (http://www.arpa.emr.it/sim/?osservazioni_e_dati/dexter).

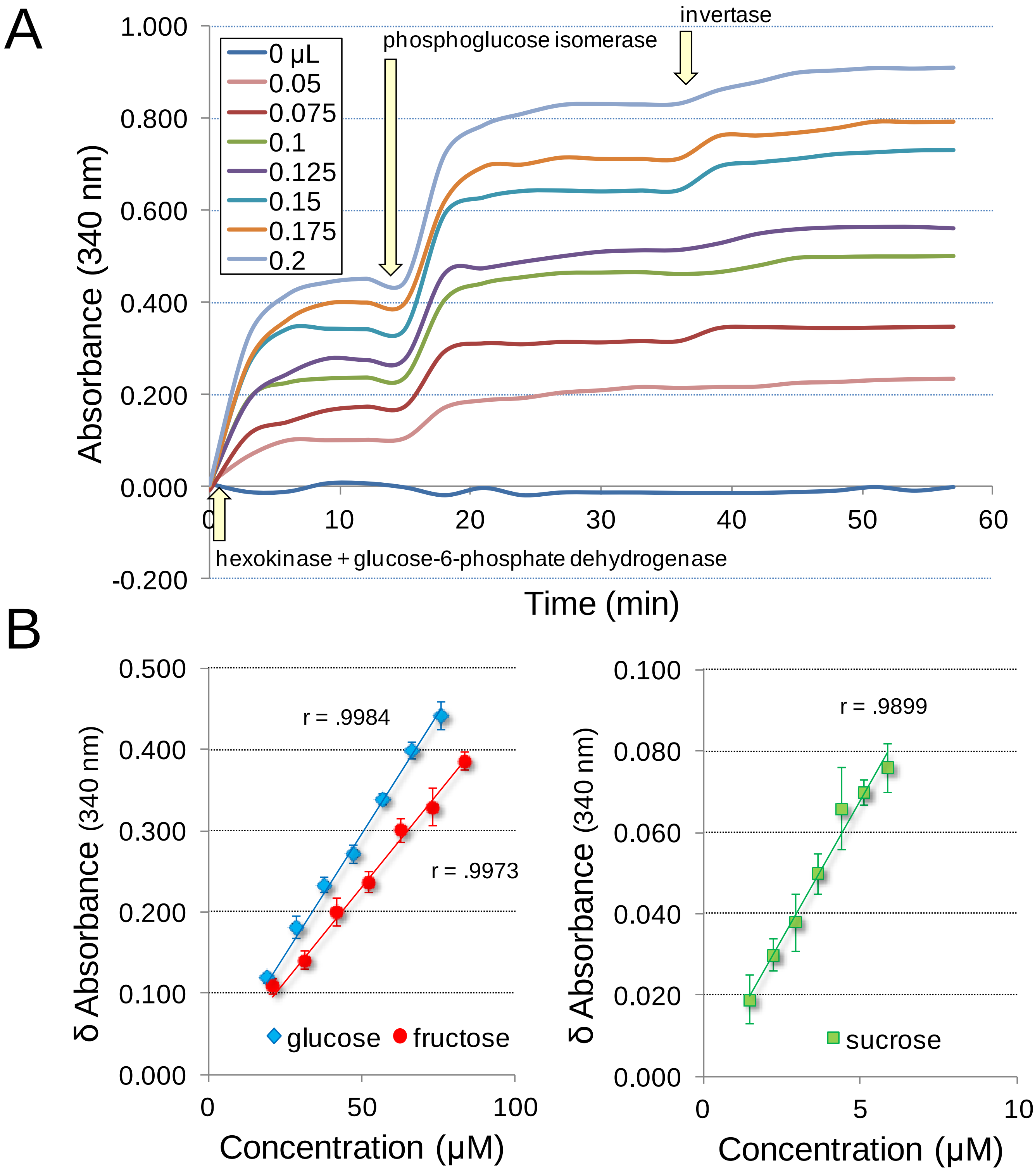

Figure S4 │ Sugar analysis. To measure glucose concentration, sample aliquots were incubated with hexokinase and glucose-6-phosphate dehydrogenase in the presence of NAD^+ and ATP. The increase of absorbance at 340 nm was followed for 15 min, until it stabilized. Fructose content was then quantified by adding phosphoglucose isomerase, following the resulting increase of absorbance for further 21 min. Sucrose concentration was finally evaluated by adding invertase, monitoring the absorbance for further 18 min. Data refer to suitable volumes, as indicated, of an artificial nectar consisting of 7.5% (w/v) of both glucose and fructose, and 1% (w/v) sucrose **(A)**. The results, expressed as the mean (± SD over 5 replicates) increase of absorbance obtained in each step, were plotted as a function of sugar concentration in the assay mixture, allowing to build the corresponding calibration curves **(B)**.

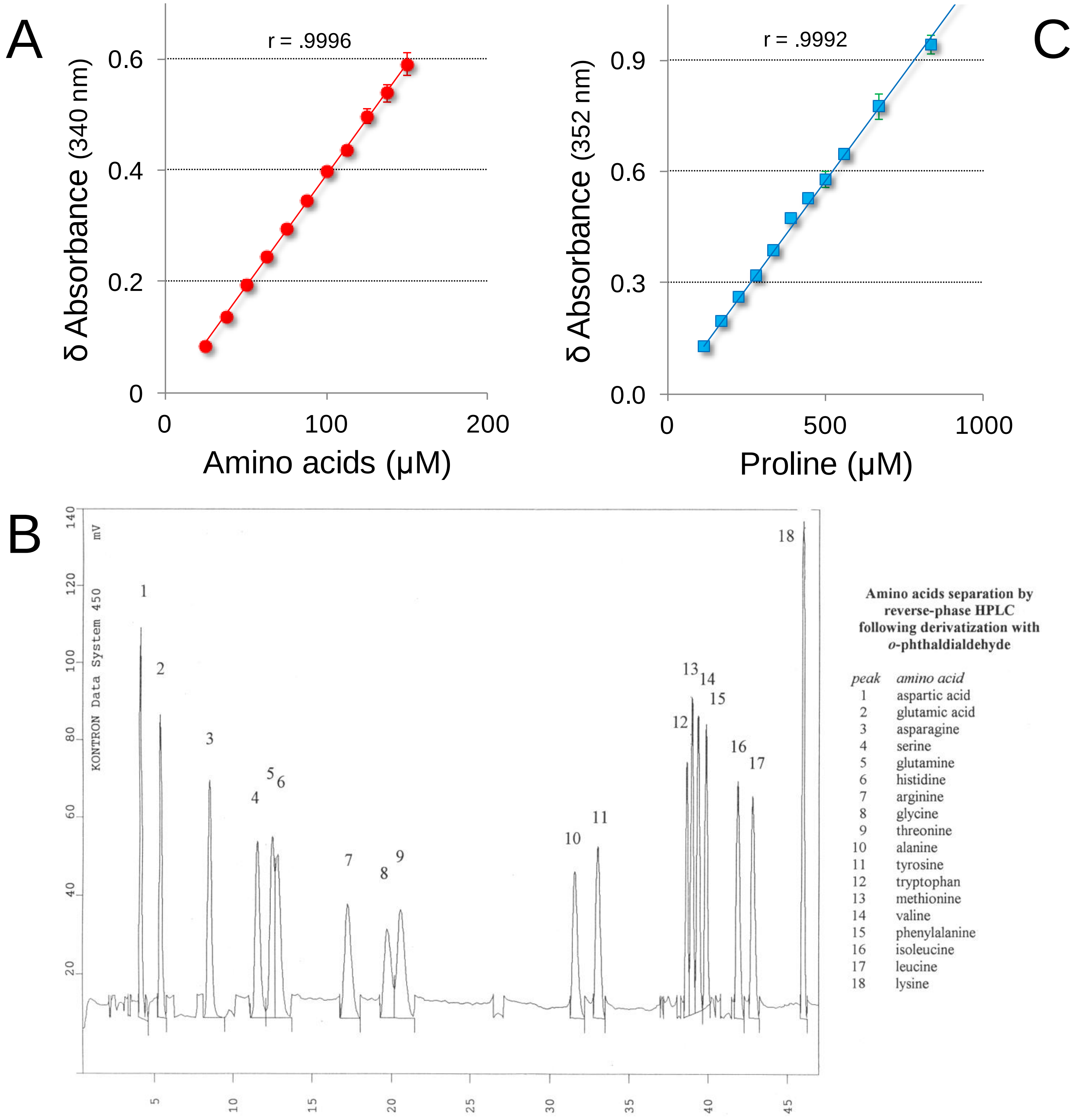

Figure S5 │ Amino acid analysis. To measure total amino acid content, sample aliquots (1 and 2 µL) were water-diluted to 50 µL, and the resulting samples were mixed with the same volume of *o*-phtaldialdehyde (*o*PDA) solution (0.5 M in 0.5 M sodium borate buffer, pH 10.0, containing 0.5 M β-mercaptoethanol and 10% [v/v] methanol). After exactly 60 sec, the increase in absorbance was measured at 340 nm. Amino acid content was estrapolated from a calibration curve obtained with a solution of all the 20 proteinogenic compounds (each at 1 mM but Glu, Asp and Asn [2 mM], and Gln [5 mM]) **(A)**. The concentration of each amino acid was quantified on 10 µL samples by RP-HPLC following derivatization with *o*PDA. Peaks were integrated by area, with variation coefficients ranging from 0.8 to 3.2%. The chromatogram refers to an equimolar mixture of amino acids, each at 0.25 mM **(B)**. Because *o*PDA does not react with proline and cysteine, the concentration of the former was measured by either RP-HPLC following derivatization with 4-dimethyl-aminoazobenzene-4'-sulfonyl chloride (not shown) or the acid ninhydrin method (Williams, I., and Frank, L. (1975). Improved chemical synthesis and enzymatic assay of δ^{1}-pyrroline-5-carboxylic acid. *Anal. Biochem.* **64**, 85–97. doi: 10.1016/0003-2697(75)90408-X). In the latter case, proline content was estrapolated from a calibration curve obtained with an authentic standard **(C)**. Free cysteine was not determined.

Table S6 │ Amino acid content in nectar of selected rapeseed varieties

Genotype	Adam		Forza		Henry		Lorenz		Milena		Nk Petrol		Shakira		Taurus	
Amino acid	mM ± SE	%	mM ± SE	%	mM ± SE	%	mM ± SE	%	mM ± SE	%	mM ± SE	%	mM ± SE	%	mM ± SE	%
Asp	0.24 ± 0.07	5.8	0.18 ± 0.05	5.8	0.22 ± 0.06	5.7	0.17 ± 0.07	5.8	0.33 ± 0.02	4.9	0.35 ± 0.11	4.3	0.14 ± 0.02	10.6	0.20 ± 0.02	5.4
Glu	0.34 ± 0.09	8.3	0.25 ± 0.11	8.0	0.27 ± 0.08	7.0	0.21 ± 0.09	7.0	0.51 ± 0.11	7.7	0.81 ± 0.24	9.9	0.11 ± 0.03	8.0	0.28 ± 0.04	7.5
Asn	0.31 ± 0.11	7.5	0.16 ± 0.03	5.1	0.30 ± 0.07	7.8	0.21 ± 0.04	6.9	0.48 ± 0.08	7.3	0.74 ± 0.12	9.1	0.10 ± 0.02	7.1	0.30 ± 0.05	8.2
Ser	0.15 ± 0.02	3.7	0.11 ± 0.05	3.6	0.16 ± 0.04	4.2	0.17 ± 0.03	5.6	0.30 ± 0.07	4.5	0.28 ± 0.07	3.4	0.05 ± 0.02	4.0	0.14 ± 0.02	3.9
Gln	1.13 ± 0.13	27.5	0.95 ± 0.26	30.6	1.08 ± 0.23	27.8	0.94 ± 0.24	31.5	1.78 ± 0.19	27.1	2.45 ± 0.55	30.2	0.44 ± 0.11	32.0	1.13 ± 0.35	30.6
His	0.49 ± 0.20	12.0	0.48 ± 0.22	15.3	0.46 ± 0.09	12.0	0.39 ± 0.12	12.9	0.81 ± 0.12	12.3	0.85 ± 0.09	10.5	0.17 ± 0.04	12.2	0.37 ± 0.11	9.9
Arg	0.03 ± 0.02	0.7	0.02 ± 0.01	0.7	0.03 ± 0.01	0.7	0.02 ± 0.01	0.7	0.04 ± 0.02	0.7	0.04 ± 0.02	0.5	0.01 ± 0.01	0.8	0.02 ± 0.01	0.6
Gly	0.09 ± 0.03	2.2	0.06 ± 0.01	2.0	0.09 ± 0.02	2.3	0.07 ± 0.03	2.4	0.12 ± 0.07	1.8	0.17 ± 0.05	2.2	0.02 ± 0.01	1.7	0.08 ± 0.03	2.2
Thr	0.11 ± 0.02	2.6	0.07 ± 0.02	2.4	0.10 ± 0.03	2.7	0.07 ± 0.01	2.2	0.17 ± 0.05	2.6	0.21 ± 0.02	2.6	0.03 ± 0.02	1.9	0.07 ± 0.03	1.9
Ala	0.28 ± 0.05	6.8	0.20 ± 0.02	6.3	0.20 ± 0.06	5.1	0.17 ± 0.04	5.8	0.42 ± 0.11	6.3	0.71 ± 0.08	8.8	0.05 ± 0.01	3.5	0.22 ± 0.05	6.0
GABA + Tyr	0.35 ± 0.04	8.4	0.20 ± 0.07	6.3	0.31 ± 0.08	8.1	0.16 ± 0.03	5.2	0.61 ± 0.19	9.3	0.43 ± 0.04	5.2	0.06 ± 0.03	4.6	0.28 ± 0.06	7.7
Trp	0.03 ± 0.01	0.8	0.01 ± 0.00	0.4	0.02 ± 0.01	0.6	0.02 ± 0.01	0.7	0.03 ± 0.01	0.5	0.03 ± 0.01	0.3	0.01 ± 0.01	0.5	0.02 ± 0.01	0.5
Met	0.01 ± 0.01	0.4	0.01 ± 0.01	0.3	0.01 ± 0.00	0.2	0.01 ± 0.01	0.2	0.02 ± 0.01	0.3	0.01 ± 0.01	0.2	0.00 ± 0.00	0.3	0.01 ± 0.01	0.2
Val	0.15 ± 0.06	3.6	0.10 ± 0.01	3.1	0.18 ± 0.05	4.6	0.12 ± 0.03	4.0	0.26 ± 0.03	4.0	0.29 ± 0.03	3.6	0.04 ± 0.02	3.2	0.14 ± 0.03	3.9
Phe	0.03 ± 0.00	0.6	0.02 ± 0.00	0.8	0.05 ± 0.03	1.3	0.04 ± 0.02	1.3	0.07 ± 0.04	1.1	0.06 ± 0.02	0.7	0.02 ± 0.01	1.2	0.04 ± 0.02	1.1
Ile	0.07 ± 0.02	1.8	0.05 ± 0.02	1.7	0.09 ± 0.02	2.2	0.06 ± 0.01	1.9	0.14 ± 0.04	2.1	0.13 ± 0.01	1.7	0.02 ± 0.00	1.7	0.07 ± 0.03	1.9
Leu	0.06 ± 0.02	1.4	0.04 ± 0.01	1.3	0.07 ± 0.02	1.9	0.04 ± 0.01	1.4	0.10 ± 0.02	1.5	0.09 ± 0.02	1.2	0.02 ± 0.01	1.6	0.06 ± 0.02	1.7
Lys	0.04 ± 0.03	1.0	0.03 ± 0.01	1.1	0.06 ± 0.01	1.5	0.04 ± 0.02	1.2	0.09 ± 0.03	1.4	0.09 ± 0.01	1.1	0.01 ± 0.01	0.9	0.04 ± 0.01	1.2
Pro	0.21 ± 0.07	5.1	0.17 ± 0.03	5.3	0.17 ± 0.03	4.4	0.10 ± 0.03	3.4	0.30 ± 0.10	4.6	0.37 ± 0.07	4.5	0.06 ± 0.02	4.3	0.20 ± 0.05	5.5
All	4.13 ± 0.61	100.0	3.12 ± 0.77	100.0	3.87 ± 0.72	100.0	2.99 ± 0.55	100.0	6.58 ± 0.96	100.0	8.12 ± 1.22	100.0	1.37 ± 0.22	100.0	3.70 ± 1.11	100.0

Single amino acids were quantified by RP-HPLC following derivatization with *o*-phtaldialdehyde. Peaks were integrated by area, with variation coefficients ranging from 0.8 to 3.2%. Since oPDA does not react with proline and cysteine, the concentration of the former was measured either by the acid ninhydrin method, or by RP-HPLC following derivatization with 4-dimethyl-aminoazobenzene-4'-sulfonyl chloride. For each sample, two technical replications were carried out. Data are means ± SE over four independent replications.

Table S7 │ Amino acid content in phloem sap of selected rapeseed varieties

Genotype	Adam		Forza		Henry		Lorenz		Milena		Nk Petrol		Shakira		Taurus	
Amino acid	mM ± SE	%	mM ± SE	%	mM ± SE	%	mM ± SE	%	mM ± SE	%	mM ± SE	%	mM ± SE	%	mM ± SE	%
Asp	3.0 ± 0.5	1.3	3.8 ± 0.5	1.9	5.8 ± 0.6	2.6	3.7 ± 0.5	1.9	4.0 ± 0.8	1.8	3.3 ± 0.2	1.5	2.5 ± 0.6	1.0	3.7 ± 0.6	2.0
Glu	10.2 ± 1.5	4.4	7.3 ± 0.8	3.6	9.3 ± 1.1	4.1	10.1 ± 1.5	5.2	12.7 ± 1.6	5.7	9.2 ± 0.8	4.2	13.0 ± 2.3	5.2	9.4 ± 1.0	5.0
Asn	5.6 ± 0.7	2.4	3.9 ± 0.2	2.0	5.2 ± 0.3	2.3	5.0 ± 0.6	2.6	5.2 ± 1.3	2.3	4.7 ± 0.6	2.2	5.8 ± 0.6	2.4	4.7 ± 0.6	2.5
Ser + Gln	141.5 ± 9.7	61.5	119.2 ± 19.6	59.8	121.1 ± 12.5	53.6	104.5 ± 1.8	53.7	124.9 ± 0.7	55.7	127.8 ± 18.9	58.3	143.9 ± 16.0	57.8	102.6 ± 9.6	54.9
His	16.5 ± 1.3	7.1	21.0 ± 3.7	10.5	24.1 ± 2.8	10.7	20.6 ± 5.5	10.6	16.1 ± 1.7	7.2	21.7 ± 4.8	9.9	24.4 ± 2.4	9.8	18.7 ± 1.9	10.0
Arg	1.6 ± 0.4	0.7	1.3 ± 0.3	0.7	1.5 ± 0.2	0.7	1.4 ± 0.1	0.7	1.8 ± 0.3	0.8	1.7 ± 0.3	0.8	1.4 ± 0.4	0.6	1.0 ± 0.2	0.6
Gly	1.3 ± 0.3	0.5	0.9 ± 0.2	0.4	1.5 ± 0.2	0.7	1.2 ± 0.2	0.6	1.4 ± 0.1	0.6	1.1 ± 0.2	0.5	1.2 ± 0.2	0.5	0.9 ± 0.1	0.5
Thr	6.4 ± 1.1	2.8	5.8 ± 0.8	2.9	7.2 ± 1.2	3.2	6.9 ± 1.2	3.5	7.0 ± 0.9	3.1	7.0 ± 0.9	3.2	7.6 ± 1.5	3.0	6.0 ± 0.5	3.2
Ala	4.2 ± 0.4	1.8	2.2 ± 0.3	1.1	2.9 ± 0.4	1.3	3.5 ± 0.5	1.8	3.8 ± 0.2	1.7	3.5 ± 0.1	1.6	4.9 ± 1.1	2.0	2.7 ± 0.3	1.4
GABA + Tyr	1.4 ± 0.1	0.6	1.1 ± 0.1	0.5	1.5 ± 0.3	0.7	1.3 ± 0.2	0.7	1.4 ± 0.2	0.6	1.1 ± 0.1	0.5	1.3 ± 0.2	0.5	1.0 ± 0.1	0.6
Trp	0.6 ± 0.1	0.2	0.6 ± 0.1	0.3	0.9 ± 0.1	0.4	0.8 ± 0.1	0.4	0.8 ± 0.2	0.4	0.8 ± 0.1	0.4	0.7 ± 0.1	0.3	0.8 ± 0.1	0.4
Met	0.4 ± 0.1	0.2	0.4 ± 0.0	0.2	0.5 ± 0.1	0.2	0.5 ± 0.1	0.2	0.7 ± 0.2	0.3	0.4 ± 0.0	0.2	0.5 ± 0.1	0.2	0.4 ± 0.1	0.2
Val	7.7 ± 1.1	3.3	6.5 ± 0.7	3.3	9.9 ± 1.7	4.4	8.7 ± 1.5	4.5	9.0 ± 2.2	4.0	8.3 ± 1.2	3.8	9.6 ± 1.7	3.9	8.1 ± 1.5	4.3
Phe	7.3 ± 1.1	3.2	6.8 ± 0.7	3.4	9.8 ± 1.8	4.3	6.8 ± 0.9	3.5	11.0 ± 2.6	4.9	6.5 ± 0.3	2.9	8.4 ± 1.8	3.4	7.1 ± 0.7	3.8
Ile	5.5 ± 0.7	2.4	4.8 ± 0.4	2.4	6.5 ± 1.2	2.9	5.0 ± 1.0	2.6	6.4 ± 1.3	2.9	5.7 ± 0.9	2.6	6.0 ± 1.1	2.4	5.2 ± 0.9	2.8
Leu	3.6 ± 0.6	1.6	3.0 ± 0.2	1.5	4.4 ± 0.9	1.9	3.5 ± 0.6	1.8	5.1 ± 1.6	2.3	3.7 ± 0.6	1.7	3.8 ± 0.9	1.5	3.6 ± 0.7	1.9
Lys	5.1 ± 0.7	2.2	4.5 ± 0.5	2.3	5.9 ± 1.1	2.6	4.1 ± 0.6	2.1	6.6 ± 1.2	2.9	5.6 ± 0.9	2.5	5.7 ± 0.9	2.3	4.4 ± 0.6	2.4
Pro	8.4 ± 1.1	3.6	6.3 ± 0.1	3.1	7.9 ± 0.3	3.5	7.1 ± 0.7	3.6	6.3 ± 1.0	2.8	7.1 ± 1.1	3.2	8.1 ± 1.2	3.3	6.7 ± 0.2	3.6
All	230.1 ± 35.4	100.0	199.3 ± 32.0	100.0	225.8 ± 21.2	100.0	194.8 ± 22.3	100.0	224.1 ± 28.4	100.0	219.3 ± 41.0	100.0	248.8 ± 26.4	100.0	186.9 ± 12.0	100.0

Single amino acids were quantified by RP-HPLC following derivatization with *o*-phtaldialdehyde. Peaks were integrated by area, with variation coefficients ranging from 0.8 to 3.2%. Since oPDA does not react with proline and cysteine, the concentration of the former was measured either by the acid ninhydrin method, or by RP-HPLC following derivatization with 4-dimethyl-aminoazobenzene-4'-sulfonyl chloride. For each sample, two technical replications were carried out. Data are means ± SE over three independent replications.
